# Depression after stoma surgery: a systematic review and meta-analysis

**DOI:** 10.1186/s12888-023-04871-0

**Published:** 2023-05-22

**Authors:** Joshua G. Kovoor, Jonathan Henry W. Jacobsen, Brandon Stretton, Stephen Bacchi, Aashray K. Gupta, Brayden Claridge, Matthew V. Steen, Ameya Bhanushali, Lorenz Bartholomeusz, Suzanne Edwards, Gayatri P. Asokan, Gopika Asokan, Amanda McGee, Christopher D. Ovenden, Joseph N. Hewitt, Markus I. Trochsler, Robert T. Padbury, Seth W. Perry, Ma-Li Wong, Julio Licinio, Guy J. Maddern, Peter J. Hewett

**Affiliations:** 1 University of Adelaide, Adelaide, South Australia Australia; 2 The Queen Elizabeth Hospital, Adelaide, South Australia Australia; 3 Royal Australasian College of Surgeons, Adelaide, South Australia Australia; 4 Royal Adelaide Hospital, Adelaide, South Australia Australia; 5 Flinders Medical Centre, Flinders University, Adelaide, South Australia Australia; 6 Health and Information, Adelaide, South Australia Australia; 7 Gold Coast University Hospital, Gold Coast, QLD Australia; 8 Glenside Health Services, Adelaide, South Australia Australia; 9 Stoma Care Services, Adelaide, South Australia Australia; 10 State University of New York Upstate Medical University, Syracuse, NY USA

**Keywords:** Depression, Stoma surgery, Mood, Patients, Nurses

## Abstract

**Background:**

Depression is the leading cause of global disability and can develop following the change in body image and functional capacity associated with stoma surgery. However, reported prevalence across the literature is unknown. Accordingly, we performed a systematic review and meta-analysis aiming to characterise depressive symptoms after stoma surgery and potential predictive factors.

**Methods:**

PubMed/MEDLINE, Embase, CINAHL and Cochrane Library were searched from respective database inception to 6 March 2023 for studies reporting rates of depressive symptoms after stoma surgery. Risk of bias was assessed using the Downs and Black checklist for non-randomised studies of interventions (NRSIs), and Cochrane RoB2 tool for randomised controlled trials (RCTs). Meta-analysis incorporated meta-regressions and a random-effects model. Registration: PROSPERO, CRD42021262345.

**Results:**

From 5,742 records, 68 studies were included. According to Downs and Black checklist, the 65 NRSIs were of low to moderate methodological quality. According to Cochrane RoB2, the three RCTs ranged from low risk of bias to some concerns of bias. Thirty-eight studies reported rates of depressive symptoms after stoma surgery as a proportion of the respective study populations, and from these, the median rate across all timepoints was 42.9% 42.9% (IQR: 24.2–58.9%). Pooled scores for respective validated depression measures (Hospital Anxiety and Depression Score (HADS), Beck Depression Inventory (BDI), and Patient Health Questionnaire-9 (PHQ-9)) across studies reporting those scores were below clinical thresholds for major depressive disorder according to severity criteria of the respective scores. In the three studies that used the HADS to compare non-stoma versus stoma surgical populations, depressive symptoms were 58% less frequent in non-stoma populations. Region (Asia–Pacific; Europe; Middle East/Africa; North America) was significantly associated with postoperative depressive symptoms (*p* = 0.002), whereas age (*p* = 0.592) and sex (*p* = 0.069) were not.

**Conclusions:**

Depressive symptoms occur in almost half of stoma surgery patients, which is higher than the general population, and many inflammatory bowel disease and colorectal cancer populations outlined in the literature. However, validated measures suggest this is mostly at a level of clinical severity below major depressive disorder. Stoma patient outcomes and postoperative psychosocial adjustment may be enhanced by increased psychological evaluation and care in the perioperative period.

**Supplementary Information:**

The online version contains supplementary material available at 10.1186/s12888-023-04871-0.

## Introduction

Depression is the leading cause of disability worldwide [1] and has an estimated lifetime prevalence of greater than 10% globally [2]. Since the disease can present in various ways [3], many who experience depressive symptoms may go undiagnosed [4]. Both psychosocial and biological stressors are implicated in the complex pathophysiology underlying depression. However, with evidence-based psychotherapy and appropriate pharmacological intervention, prognosis can be favourable [5]. To increase the likelihood of positive outcomes, low socioeconomic and other marginalized populations experiencing depressive symptoms must be identified and provided effective care. This is of particular importance as an association between depression and multiple forms of inequality, including gender [6], and socioeconomic position has been observed [7]. Further, to avoid healthcare inequity, it is imperative that when patients with stomas are from potentially marginalised populations, such as those of minority ethnic backgrounds, they have their sociocultural needs addressed [8], and receive comprehensive and integrative care within a biopsychosocial model [9].

Surgery is a common medical need, with the average person undergoing multiple operations across their lifetime [10]. Patients that experience considerable change in body image or functional capacity following surgery can develop depressive symptoms in the postoperative period [4]. Stoma surgery is frequently conducted, most commonly to treat either cancer, inflammatory bowel disease, or diverticular disease. As stoma surgery and the resultant ostomy bag represent both psychological and biological stressors, the mental health of these patients may be affected during this postoperative psychosocial adjustment period [11, 12]. It is estimated that around 25% of stoma patients experience clinically significant psychological symptoms after surgery [12].

A comprehensive analysis of the rates and factors associated with risk of increased depressive symptoms across the international literature of depressive symptoms after stoma surgery has not been conducted. In particular, descriptions of clinical severity and perioperative change in depressive symptoms, and effect of age, sex, geographic region, type of stoma, stoma permanency, surgical pathology, and postoperative time, have not been investigated within a single review. Accordingly, to inform the biopsychosocial clinical care of patients undergoing stoma surgery and living with stomas worldwide, we performed this systematic literature review and meta-analysis aiming to characterise rates of depressive symptoms after stoma surgery, describe clinical severity and perioperative change in these symptoms, and also identify potential predictive factors such as age, sex, geographic region, type of stoma, stoma permanency, surgical pathology, and time after surgery.

## Methods

The methods protocol for this study was generated prior to its conduct. The protocol was prospectively submitted for registration with PROSPERO (number CRD42021262345), and follows the Preferred Reporting Items for Systematic Reviews and Meta-Analyses 2020 (PRISMA 2020) [13] and Meta-analyses Of Observational Studies in Epidemiology (MOOSE) [14] reporting guidelines.

### Search strategy and selection criteria

The population, intervention, comparator group, outcome (PICO) framework was used to formulate the research question and inclusion criteria [15]. The population comprised patients of all ages undergoing surgery resulting in a stoma in any country. The intervention was surgery producing a stoma. There was no overall comparator group, however comparisons were made between various sub-populations of stoma patients. Outcomes included measures of depressive symptoms. Editorials, perspectives, letters, and conference abstracts were considered inappropriate for analysis, and were excluded.

PubMed (incorporating MEDLINE), Embase, CINAHL, and the Cochrane Library were searched from database inception to 6 March 2023 for studies of any design and in any setting that reported rates of depressive symptoms after surgery resulting in a stoma. Publications from any country were included. The search strategies were designed to include DSM-5 requirements for major depressive disorder of either depressed mood or anhedonia [3], and are detailed within Additional file 1: Appendix 1. Searches were not limited by language and no publication restrictions were implemented. During the process of searching, 14 full-texts could not be obtained.

### Data extraction

Two reviewers independently screened titles and abstracts, reviewed full texts, and extracted data using a standard extraction form. Screening of titles and abstracts was facilitated via a web application (Rayyan, Qatar Computing Research Institute, Ar-Rayyan, Qatar) [16]. Disagreements were resolved by consensus. The extracted data included research design, study setting, population characteristics, intervention characteristics, comparator characteristics, timeframe for follow-up, quantitative and qualitative outcomes, source of funding and reported conflicts of interest, methodological quality information, and other information relevant to the review questions. Data were synthesised in narrative and tabular formats. The primary outcome was rates of depressive symptoms after stoma surgery. Effect on depressive symptoms was investigated for age, sex, region, before versus after stoma surgery, stoma versus no stoma populations, colostomy versus ileostomy, permanent versus temporary stoma, surgical pathology, and time after surgery.

### Data analysis

Data analyses were performed using Stata Statistical Software: Release 15.1 College Station, TX: StataCorp LP. To evaluate heterogeneity, we used the I^2^ statistic (with I^2^ > 50% indicating significant heterogeneity) and Cochran’s Q *p* value (with *p* < 0.05 indicating significant heterogeneity). A random-effects model was used throughout. A *p* value of < 0.05 denoted statistical significance. A Funnel plot was constructed for each variable to test for publication bias. An Egger’s Test was performed for each variable to test for small study effects. A variable was included in the meta-analysis if ≥ 2 articles meeting inclusion criteria reported sufficient data for that variable.

Prevalence of depressive symptoms (with 95% confidence intervals (CIs)) were calculated for each study, and all studies were combined within a Forest plot. Predictors of mean age, male sex, and region of procedure (with 95% confidence intervals) were presented in a Forest plot. Within this study, regions were split into four groups: Asia–Pacific; Europe; Middle East/Africa; and North America. Meta-regressions were performed incorporating the prevalence of depression versus these respective predictors. Odds of experiencing depressive symptoms before and after stoma surgery, and for stoma versus non-stoma patients, were calculated for each respective study (with 95% CIs), then all respective studies were combined within a Forest plot. Mean differences regarding depressive symptoms for stoma versus non-stoma patients (measured via the Hospital Anxiety and Depression Scale (HADS)) [17], and colostomy versus ileostomy patients, were calculated for each respective study (with 95% CIs), and all respective studies were combined within a Forest plot.

Methodological quality was independently assessed by two reviewers using validated tools. The Downs and Black checklist [18] was used for risk of bias assessment for included non-randomised studies of interventions (NRSIs). This checklist evaluates risk of confounding and selection bias, methods used to ascertain exposures and outcomes, and selection of the reported results from among multiple measurements or analyses of specified outcomes. The subsections within this checklist incorporate measurements of study reporting, external validity, internal validity, bias, confounding, and statistical power. Within the original version of the Downs and Black checklist that was used, no specific scoring system or cut-offs are specified, with the independent reviewers conducting the critical appraisal left to make an overall assessment of the study’s methodological quality and risk of bias based on a total score out of 32 [18]. Version 2 of the Cochrane tool for assessing risk of bias in randomised trials [19] was used to critically appraise any included randomised controlled trials (RCTs).

## Results

### Study characteristics

**Fig. 1:**
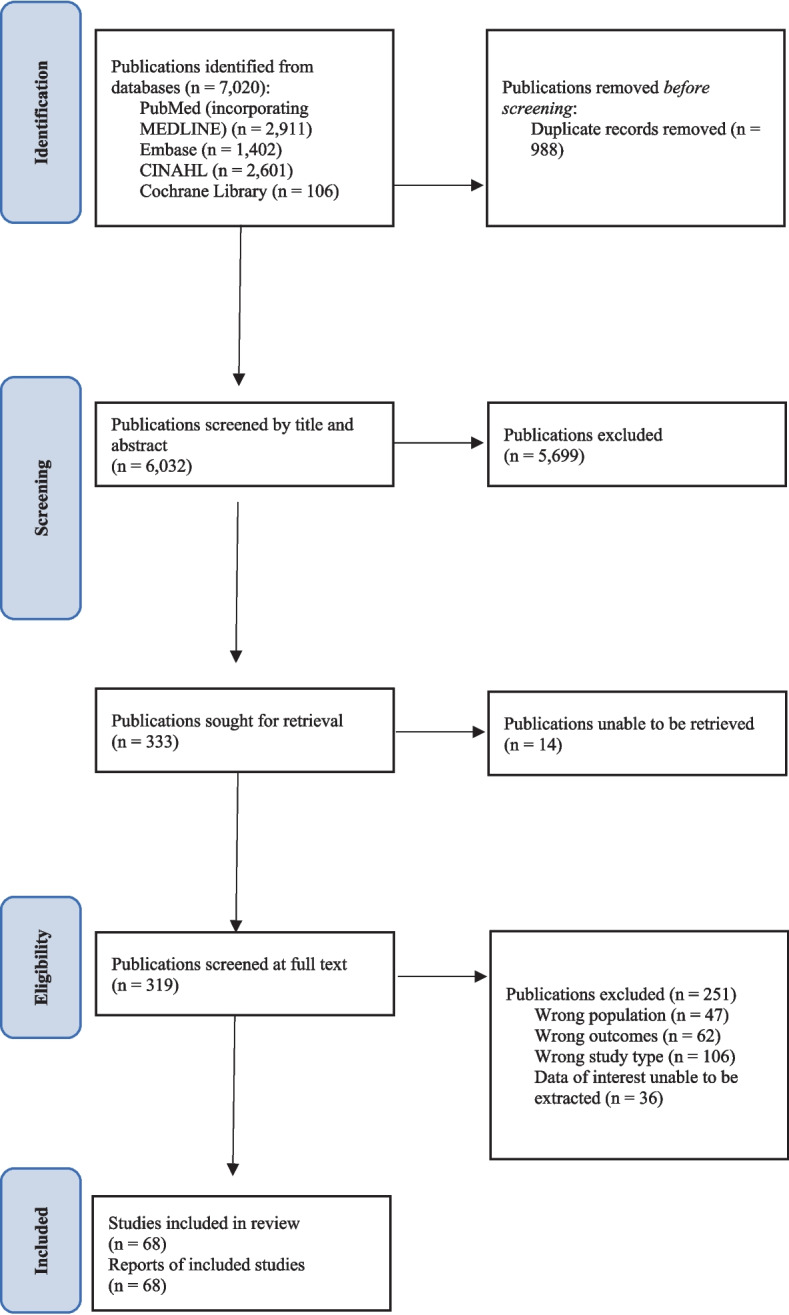
Study selection

Searches identified a total of 7,020 records (6,032 unique reports), from which 333 full-text articles were retrieved and 68 of these studies were included (Fig. 1). Median sample size across all studies was 66 (IQR: 38.8–187). A list of studies excluded at full-text review, with justification of exclusion for each potentially relevant study, can be found in Additional file 1: Appendix 2. The characteristics of the included studies are outlined in Table 1.

**Table 1:** Characteristics of included studies reporting depressive symptoms after stoma surgery

Author	Year	Country	Design	Sample Size	Measure of Depressive Symptoms	Pathology	Stoma Type(s)
Abdalla [20]	2016	USA	Survey	398	PROMIS	IBD	NS
Ananthakrishnan [21]	2013	USA	Retrospective cohort	158	ICD-9	IBD	NS
Anaraki [22]	2012	Iran	Survey	102	Non-validated	Cancer, IBD, polyps, necrosis, trauma, peritonitis, obstruction, fistula, congenital, radiation	Ileostomy, colostomy, urostomy
Anaraki [23]	2012 (2)	Iran	Survey	102	Non-validated	Cancer, IBD, polyps, necrosis, trauma, peritonitis, obstruction, fistula, congenital, radiation	Ileostomy, colostomy, urostomy
Armbruster [24]	2018	USA	Survey	41	Center for Epidemiologic Studies-Depression Scale	Cancer	Colostomy
Bahayi [25]	2018	Turkey	Retrospective case–control	50	Beck Depression Inventory	Colorectal cancer	Colostomy, ileostomy
Barisic [26]	2011	Serbia	Prospective cohort	45	Depression scale of Faecal Incontinence quality of life scale	Rectal cancer	Ileostomy
Bau [27]	2001	France	Survey	19	Non-validated	Fecal incontinence	Urostomy
Blackwell [28]	2020	UK	Retrospective cohort	401	New antidepressant use postoperatively	Crohn’s disease	NS
Bossema [29]	2011	Netherlands	Survey	62	Hospital Anxiety and Depression Scale	Rectal cancer	NS
Bullen [30]	2012	Australia	Comparative cohort	22	Hospital Anxiety and Depression Scale	Colorectal cancer, IBD	NS
Chaudhri [31]	2005	UK	Randomised Controlled trial	21	Hospital Anxiety and Depression Scale	NS	Ileostomy, colostomy
Chen [32]	2013	USA	Survey	22	University of Washington Quality of Life Instrument	Head and neck cancer	Tracheostomy or stoma dependent
Coggrave [33]	2012	UK	Survey	91	Hospital Anxiety and Depression Scale	Spinal cord injury	NS
Colquhoun [34]	2006	USA	Comparative cohort	39	Depression Scale of Fecal Incontinence Quality of Life Score	Fecal incontinence	Colostomy
Cotrim [35]	2008	Portugal	Comparative cohort	46	Hospital Anxiety and Depression Scale	Colorectal cancer	NS
Davidson [36]	2016	Ireland	Survey	256	Non-validated	Cancer, IBD	Ileostomy
Davis [37]	2020	India	Survey	55	Non-validated	Cancer and NS	Ileostomy, colostomy
Geng [38]	2017	China	Survey	729	Hospital Anxiety and Depression Scale	NS	Colostomy, ileostomy, urostomy
Gonzalez [39]	2016	Sweden	Survey	545	Non-validated	Rectal cancer	Colostomy
Grant [40]	2011	USA	Survey and qualitative	51	Non-validated	Colorectal cancer	NS
Holzer [41]	2005	International	Survey	257	Depression Scale of Faecal Incontinence Quality of Life Score	Rectal cancer	Colostomy
Hong [42]	2014	South Korea	Survey	65	Beck Depression Inventory	Cancer, IBD, intestinal obstruction, colon perforation	Ileostomy, colostomy
Hornbrook [43]	2011	USA	Survey	284	Psychological domain of City of Hope Quality of Life	Colorectal cancer	NS
Iqbal [44]	2018	UK	Survey	24	Hospital Anxiety and Depression Scale	Chronic constipation	Ileostomy, colostomy
Jayarajah [45]	2017	Sri Lanka	Survey	41	Patient Health Questionnaire-9	NS	Ileostomy, colostomy
Jayarajah [46]	2017 (2)	Sri Lanka	Survey	43	Non-validated	NS	Ileostomy, colostomy
Jin [47]	2019	China	Survey	67	Hospital Anxiety and Depression scale	Rectal cancer	Colostomy
Karakayali [48]	2016	Turkey	Comparative cohort	21	Fecal Incontinence Quality of Life	Radiation-induced recto-vaginal fistula	Ileostomy
Kaltikangas-Jaryinen [49]	1983	Finland	Prospective cohort	66	Beck Depression Inventory	Colorectal cancer, ulcerative colitis	Colostomy
Kaltikangas-Jaryinen [50]	1984	Finland	Prospective cohort	66	Beck Depression Inventory	Colorectal cancer, ulcerative colitis	Colostomy, ileostomy
Ketterer [51]	2021	Australia	Survey	280	Non-validated	Bowel cancer and NS	Colostomy, ileostomy, urostomy
Knowles [52]	2013	Australia	Survey	83	Hospital Anxiety and Depression Scale	IBD	Colostomy, ileostomy
Knowles [53]	2013 (2)	Australia	Survey	31	Hospital Anxiety and Depression Scale	Crohn’s disease	NS
Knowles [54]	2014	Australia	Survey	150	Hospital Anxiety and Depression scale	IBD, cancer, diverticular disease, and NS	Ileostomy, colostomy
Koc [55]	2022	Turkey	Randomised Controlled Trial	214	Hospital Anxiety and Depression Scale	IBD, cancer, polyposis syndromes, perianal benign diseases	Ileostomy, colostomy
Krouse [56]	2009	USA	Survey	246	Non-validated	Rectal cancer	Colostomy, ileostomy
Krouse [57]	2016	USA	Survey	38	Hospital Anxiety and Depression scale	Rectal or bladder cancer	Colostomy, ileostomy, urostomy
Lamb [58]	2019	UK	Survey	19	Non-validated	NS	NS
Leminski [59]	2021	Poland	Comparative cohort	95	Hospital Anxiety and Depression Scale	Bladder cancer	Uterostomy, ileostomy
Lim [60]	2019	Singapore	Randomised Controlled Trial	24	Hospital Anxiety and Depression Scale	Colorectal cancer	Ileostomy, colostomy
Liu [61]	2021	China	Case–control	63	Hamilton Depression Rating Scale	Colorectal cancer	Colostomy
Lowe [62]	2019	UK	Survey	92	Patient Health Questionnaire-9	NS	Colostomy, ileostomy, urostomy
MacDonald [63]	1985	UK	Qualitative study	265	Interviews	Rectal cancer	Colostomy
Mohamed [64]	2021	USA	Qualitative study	30	Interviews	Bladder and colorectal cancer	Colostomy, ileostomy, urostomy
Mols [65]	2014	Netherlands	Survey	407	Hospital Anxiety and Depression Scale	Rectal cancer	NS
Norton [66]	2005	UK	Survey	66	Hopital Anxiety and Depression Scale	Fecal incontinence	Colostomy
Park [67]	2018	South Korea	Survey	217	Center for Epidemiological Studies Depression Scale	NS	NS
Portier [68]	2005	France	Observational cohort	18	Depression scale of Fecal Incontinence Quality of Life	Rectal and anal cancer	Colostomy
Powell-Chandler [69]	2020	UK	Survey	371	Patient Health Questionnaire-9	NS	NS
Rafiei [70]	2017	Iran	Survey	70	Depression, Anxiety, Stress Scale 21	NS	Colostomy, ileostomy
Rafiei [71]	2019	Iran	Survey	70	Depression, Anxiety, Stress Scale 21	NS	Colostomy, ileostomy
Ramer [72]	1992	USA	Observational cohort	12	Depression dimension of Brief Symptom Inventory	NS	Colostomy
Reese [73]	2014	USA	Survey	25	Center for Epidemiologic Studies Depression Scale-Short Form	Colorectal cancer	Colostomy, ileostomy
Repic [74]	2018	Serbia	Survey	67	Depression scale of Quality of Life Questionnaire for a Patient with an Ostomy	NS	Colostomy, ileostomy, urostomy
Richbourg [75]	2007	USA	Survey	43	Non-validated	NS	Colostomy, ileostomy, urostomy
Rud [76]	2022	Denmark	Survey	178	Major Depression Inventory	IBD, colorectal cancer, chronic constipation, surgical complications, ischemic bowel disease, familial adenomatous polyposis, diverticulitis, incarceration	Ileostomy
Sceats [77]	2020	USA	Retrospective cohort	1965	ICD-9 and ICD-10 codes	IBD	NS
Sharpe [78]	2011	Australia	Comparative cohort	34	Hospital Anxiety and Depression Scale	Colorectal cancer	NS
Shrestha [79]	2022	Nepal	Prospective cohort	116	Hospital Anxiety and Depression Scale	Cancer, IBD, trauma, complication of radiotherapy, bowel obstruction, intestinal tuberculosis	Ileostomy, colostomy, urostomy
Sivero [80]	2022	Italy	Retrospective cohort	12	Non-validated	Colorectal cancer	Colostomy
Song [81]	2020	China	Comparative cohort	148	Hospital Anxiety and Depression Scale	Colorectal cancer	NS
Ssewanyana [82]	2021	Uganda	Survey	51	Patient Health Questionnaire-9	Intestinal obstruction, colorectal cancer, penetrating abdominal trauma, aganglionic colon and NS	Colostomy, ileostomy
Thomas [83]	1987	UK	Observational interview	68	Non-validated	Bowel cancer, IBD, diverticular disease	NS
Wang [84]	2018	China	Comparative cohort	231	Non-validated	Rectal cancer	Sigmoidostomy
Williams [85]	1983	UK	Comparative cohort	38	Leeds Self Assessment of Depression Scale	Rectal cancer	Colostomy
Wirsching [86]	1975	Germany	Case–control	214	Non-validated	Rectal cancer	Colostomy
Zewude [87]	2021	Ethiopia	Prospective cohort	64	City of Hope Quality of Life – Ostomy Questionnaire	Cancer, obstruction, trauma	Colostomy, ileostomy

*IBD* Inflammatory bowel disease, *NS* Not specified

### Depressive symptoms after stoma surgery

Of the included studies, 44 reported rates of depressive symptoms after stoma surgery as a proportion of the respective study populations. Median sample size within these studies was 76.5 (IQR: 43–214.8). From these populations, the median rate of depressive symptoms after stoma surgery was 42.9% (IQR: 24.2–58.9%) across all timepoints after surgery. Overall results of the meta-analyses that were conducted, including the Egger’s tests for small study effects and meta-regressions, are summarised in Table 2.

**Table 2:** Results of Egger’s test for small study effects and meta-regressions

Test	Dataset	Outcome	Predictor	Comparison	Mean difference (95% CI)	Comparison *P* value	Global *P* value
Egger	Age	Prevalence of depressive symptoms					0.635
Meta-regression	Age	Prevalence of depressive symptoms	Age		-0.003 (-0.128, 0.007)		0.592
Egger	Age	Age					0.001
Egger	Gender	Prevalence of depressive symptoms					0.930
Meta-regression	Gender	Prevalence of depressive symptoms	Proportion Male		0.410 (-0.034, 0.854)		0.069
Egger	Gender	Gender					0.224
Egger	Region	Prevalence of depressive symptoms					0.041
Meta-regression	Region	Prevalence of depressive symptoms	Region	Europe vs Asia–Pacific	0.037 (-0.115, 0.188)	0.629	0.002
				Middle East / Africa vs Asia–Pacific	0.364 (0.162, 0.566)	0.001	
				North America vs Asia–Pacific	-0.033 (-0.220, 0.154)	0.722	
Egger	Before versus after stoma surgery	Odds of depressive symptoms					0.229
Egger	Other: Stoma versus No stoma	Odds of depressive symptoms					0.352
Egger	HADS: Stoma versus No stoma	Mean difference of depressive symptoms					0.461
Egger	Colostomy versus Ileostomy	Mean difference of depressive symptoms					Did not converge

Depressive symptoms were measured using both validated and non-validated tools across the 68 included studies. The most common validated measures that were used were the Hospital Anxiety and Depression Score (HADS, total score of 21) [17], the Beck Depression Inventory (BDI, total score of 60) [88], and the Patient Health Questionnaire-9 (PHQ-9, total score of 27) [89]; these were used to measure postoperative depressive symptoms in 17, 4, and 4 studies, respectively. Of the included studies, 17 studies measured postoperative depressive symptoms using the HADS and reported extractable mean or median scores across the study cohorts [29–31, 33, 35, 38, 44, 47, 52–54, 57, 60, 65, 66, 78, 81]. For the 14 studies that reported mean HADS scores, the median reported mean HADS score was 5.1 (IQR: 4.5–7.0) [29–31, 35, 38, 44, 47, 52–54, 57, 60, 78, 81]. The other three studies utilising the HADS reported median scores, and the median reported median HADS score was 3 (IQR: 2.75–3) [33, 65, 66]. These HADS scores are within the normal range (0–7) for the HADS scoring criteria, and thus did not meet the clinical threshold for major depressive disorder [17]. Of the included studies, four used the PHQ-9 as a measure of depressive symptoms, however none reported raw PHQ-9 scores, merely rates of depressive symptoms within the study population [45, 62, 69, 82]. Of the four included studies that used the BDI, all reported mean scores [25, 42, 49, 50]. For these, the median reported mean BDI score was 10.0 (IQR: 5.7–14.0), which was within the normal range of the scoring criteria (0–10), whereas the upper quartile falls into the mild mood disturbance range (11–16) [88].

### Effect of age

**Fig. 2:**
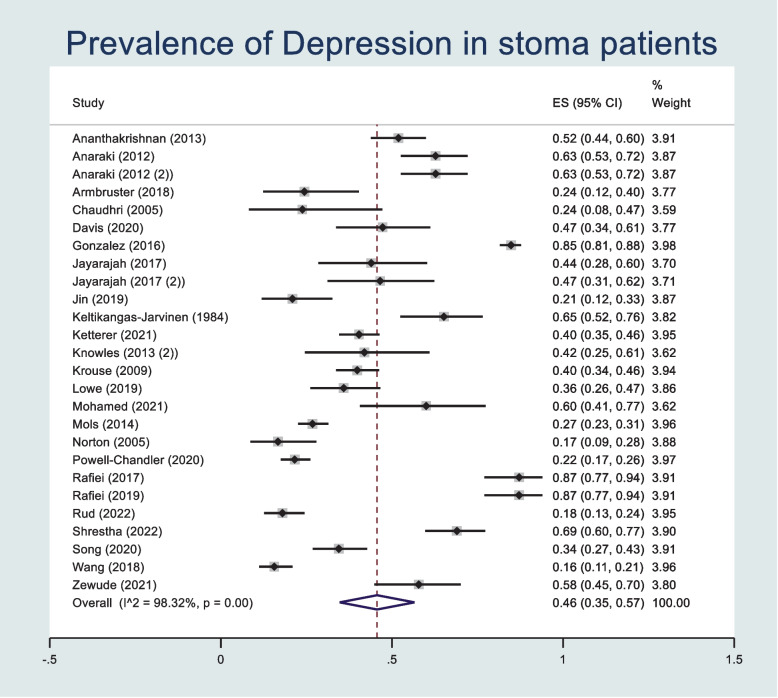
Forest plot showing prevalence of depressive symptoms after stoma surgery in studies reporting patient age

**Fig. 3:**
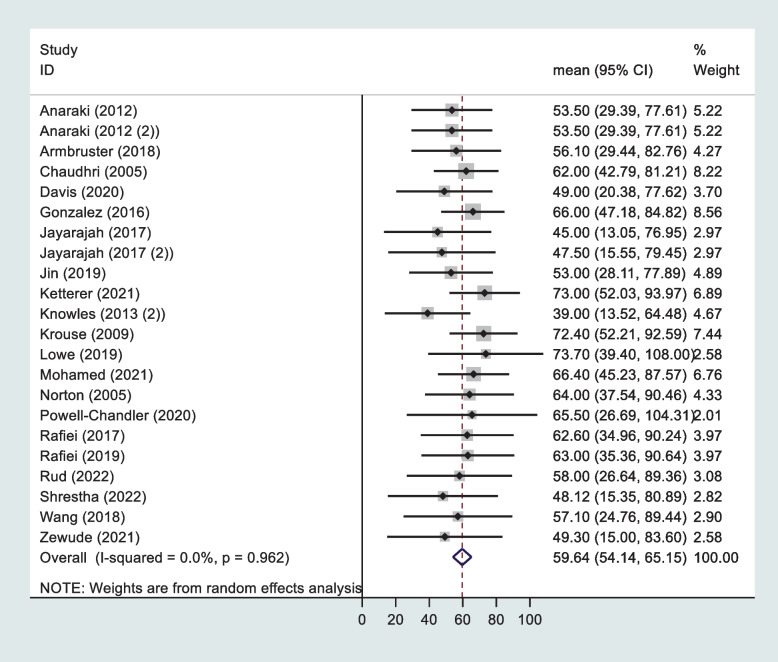
Forest plot summarising mean age and standard deviations in studies with data regarding effect of age on depressive symptoms after stoma surgery

The prevalence of postoperative depressive symptoms was pooled across 27 studies reporting extractable data regarding age using a random effects meta-analysis model. Heterogeneity in the study estimates was assessed using the I-squared statistic (98.3%) and Cochran's Q *p* value (< 0.001) which showed significant heterogeneity. The overall mean prevalence of depressive symptoms within these studies was 0.46 (95% confidence interval (CI): 0.35, 0.57) (Fig. 2). A Funnel plot shows possible publication bias (Supplementary Fig. 1), however the Egger’s Test does not suggest small study effects (*p* = 0.635). Means and standard deviations of age were pooled across 22 studies using a random effects meta-analysis model (Fig. 3). Heterogeneity in the study estimates was assessed using the I-squared statistic (0%) and Cochran's Q *p* value (0.962) which showed no heterogeneity. The overall mean age is 59.6 (95% CI: 54.1, 65.2). A Funnel plot indicated publication bias and an Egger test indicated small study effects (*p* = 0.001) (Supplementary Fig. 2). A meta-regression was performed to assess the association between prevalence of postoperative depressive symptoms and age as a predictor. No statistically significant association was found (*p* = 0.592).

### Effect of sex

**Fig. 4:**
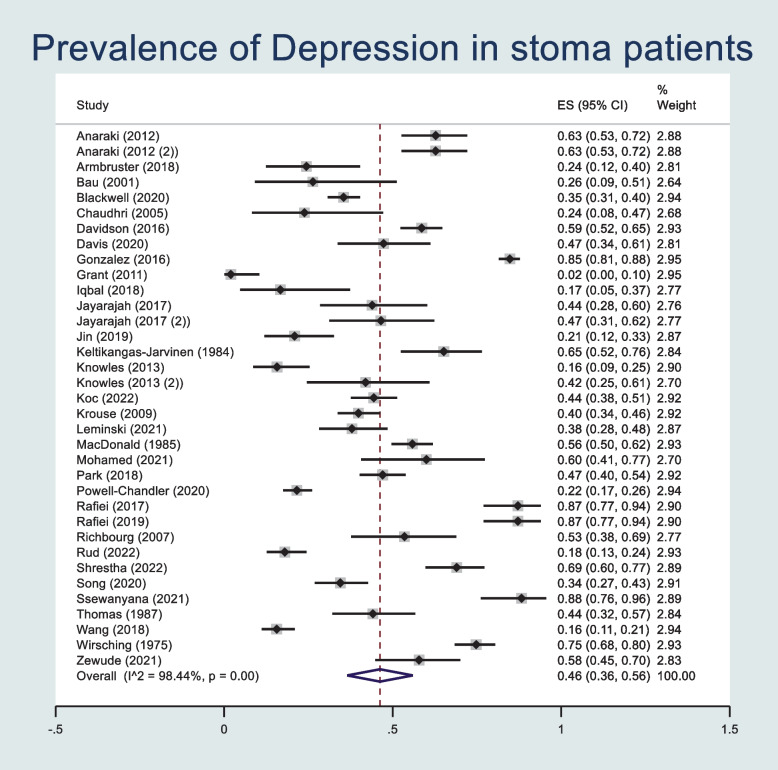
Forest plot showing prevalence of depressive symptoms after stoma surgery in studies reporting patient sex

The prevalence of postoperative depressive symptoms was pooled across 35 studies reporting extractable data regarding sex using a random effects meta-analysis model. Heterogeneity in the study estimates was assessed using the I-squared statistic (98.44%) and Cochran’s Q *p* value (< 0.001) which showed significant heterogeneity, however this was less relevant as a random effects model was used. The overall mean prevalence of depressive symptoms within these studies was 0.46 (95% confidence interval (CI): 0.36, 0.56) (Fig. 4). A Funnel plot shows possible publication bias (Supplementary Fig. 3), however Egger’s Test does not suggest small study effects (*p* = 0.930). Prevalence of male sex was pooled across the 35 studies using a random effects meta-analysis model (Supplementary Fig. 4). Heterogeneity in the study estimates was assessed using the I-squared statistic (91.21%) and Cochran’s Q *p* value (< 0.001) which showed significant heterogeneity, however this was less relevant as a random-effects model was used. The overall mean proportion of male sex within the study populations was 0.55 (95% CI: 0.50, 0.60). A Funnel plot indicated publication bias and an Egger test indicated no small study effects (*p* = 0.226) (Supplementary Fig. 5). A meta-regression was performed to assess the association between prevalence of postoperative depressive symptoms and sex as a predictor. No statistically significant association was found (*p* = 0.069).

### Effect of geographic region

The prevalence of postoperative depressive symptoms was pooled across 44 studies reporting extractable data regarding region using a random effects meta-analysis model. Heterogeneity in the study estimates was assessed using the I-squared statistic (98.6%) and Cochran’s Q *p* value (< 0.001) which showed significant heterogeneity, however this was less relevant as a random effects model was used. The overall mean prevalence of depressive symptoms within these studies was 0.44 (95% confidence interval (CI): 0.36, 0.52) (Supplementary Fig. 6). A Funnel plot shows possible publication bias (Supplementary Fig. 7) and Egger’s Test does not suggest small study effects (*p* = 0.041). Meta-regressions were performed to assess the association between prevalence of depressive symptoms and region as a predictor. Within this, study regions were split into four groups: Asia–Pacific; Europe; Middle East/Africa; and North America. There was a statistically significant association between region and postoperative depressive symptoms (*p* = 0.002). When comparing Europe versus Asia–Pacific regions, the mean difference was 0.037 (-0.115, 0.188), and this was not statistically significant (*p* = 0.629). When comparing Middle-East/Africa versus Asia–Pacific regions, the mean difference was 0.364 (0.162, 0.566), and this was statistically significant (*p* = 0.001). When comparing North America versus Asia–Pacific regions, the mean difference was -0.033 (-0.220, 0.154), and this was not statistically significant (*p* = 0.722).

### Before versus after stoma surgery

The odds of experiencing depressive symptoms before versus after stoma surgery were pooled across four studies providing extractable data using a random-effects meta-analysis model. Within these studies, follow-up ranged up to one year after stoma surgery [47]. Heterogeneity in the study estimates was assessed using the I-squared statistic (0%) and Cochran’s Q *p* value (0.592) which showed no heterogeneity. The overall difference in odds of experiencing depressive symptoms was 24% greater after stoma surgery versus before (1.24, 95% CI: 0.95, 1.62) (Supplementary Fig. 8). A Funnel plot showed no possibility of publication bias (Supplementary Fig. 9), and an Egger’s test indicated no small study effects (0.229).

### Stoma versus non-stoma populations

Across four eligible studies, stoma and non-stoma populations were directly compared with regards to odds of experiencing postoperative depressive symptoms. Heterogeneity in the study estimates was assessed using the I-squared statistic (79%) and Cochran’s Q *p* value (0.003) which showed significant heterogeneity, however this was less relevant as a random-effects model was used. The overall difference in odds of experiencing postoperative depressive symptoms was 27% greater in non-stoma surgical population versus stoma surgical populations (1.27, 95% CI: 1.14, 1.40) (Supplementary Fig. 10). A Funnel plot showed a small possibility of publication bias (Supplementary Fig. 11), and an Egger’s test indicated no small study effects (0.352).

Mean differences in postoperative depressive symptoms, as measured by the HADS [17] in stoma versus non-stoma populations were pooled across three studies providing extractable data using a random-effects meta-analysis model. Heterogeneity in the study estimates was assessed using the I-squared statistic (74.6%) and Cochran’s Q *p* value (0.02) which showed significant heterogeneity, however this was less relevant as a random-effects model was used. The overall mean difference in postoperative depressive symptoms was 58% less in non-stoma surgical population versus stoma surgical populations (0.42, 95% CI: 0.20, 0.65) (Supplementary Fig. 12). A Funnel plot showed no possibility of publication bias (Supplementary Fig. 13), and an Egger’s test indicated no small study effects (0.461).

### Colostomy versus Ileostomy

Mean differences in postoperative depressive symptoms in colostomy versus ileostomy populations were pooled across two studies providing extractable data using a random-effects meta-analysis model. Heterogeneity in the study estimates was assessed using the I-squared statistic (0%) and Cochran’s Q *p* value (0.804) which showed no heterogeneity. The overall mean difference in postoperative depressive symptoms was 51% less in ileostomy populations versus colostomy populations (0.49, 95% CI: 0.15, 0.84) (Supplementary Fig. 14). A Funnel plot showed no possibility of publication bias (Supplementary Fig. 15), and an Egger’s test did not converge.

### Permanent versus temporary stoma

Amongst the included studies, four compared depressive symptoms in cohorts of permanent versus temporary stomas, all using different measures of depression. Blackwell et al. found higher rates of depressive symptoms requiring antidepressant medication in those with permanent stomas versus those with temporary stomas (37.1% versus 33.5%) [28]. Davis et al. found that those with permanent stomas had significantly greater depressive symptom severity than those with temporary stomas (*p* = 0.023) [37]. Hong et al. compared BDI scores in the two populations, however did not find a statistically significant difference (*p* = 0.276) [42]. In contrast, Knowles et al. found greater depression severity scores in those with a temporary stoma versus those with a permanent stoma, however it was not stated whether this difference was statistically significant [53].

### Effect of surgical pathology

Of the included studies, three compared depressive symptoms in populations with different surgical pathologies [20–22]. Abdalla et al. measured depressive symptoms using the Patient-Reported Outcomes Measurement Information System (PROMIS) in stoma patients who underwent surgery for Crohn’s disease that was active versus in remission, and found greater depression severity in those with active disease [20]. Ananthakrishnan et al. compared rates of depression in cohorts of Crohn’s disease and ulcerative colitis patients, and found higher rates in those with Crohn’s disease (65.9% versus 47%, respectively) [21]. Anaraki et al. compared rates of depression in cancer versus non-cancer patients, and did not find a statistically significant difference (*p* = 0.19) [22].

### Effect of time after surgery

Of the included studies, three reported longitudinal data characterising changes in depressive symptoms with time after surgery [24, 30, 79]. Armbruster et al. measured depressive symptoms using the Center for Epidemiologic Studies-Depression Scale, whereas Bullen et al. and Shrestha utilised the HADS. Armbruster et al. reported depressive symptoms preoperatively, 6 months postoperatively, and 12 months postoperatively in a population of women undergoing pelvic exenterations, and found a change with time that was not significant (*p* = 0.78) over the three respective time points [24]. Bullen et al. reported depressive symptoms preoperatively and 3 months postoperatively in a population of patients undergoing surgery for colorectal cancer or inflammatory bowel disease, and found that stoma patients experienced significantly greater levels of depressive symptoms throughout the study and increased depression severity with time [30]. Shrestha et al. investigated a range of patients with a stoma and found that proportions of abnormal depressive symptoms within the study population decreased relative to their time from stoma surgery: 71.4% at 2 months to 1 year, 65.1% at 2–5 years, and 58.3% at greater than 6 years [79].

### Risk of bias

The 65 included NRSIs that were critically appraised using the Downs and Black checklist [18] were critically appraised by the two independent reviewers to be of low to moderate methodological quality overall. The individual breakdown of these scores can be found in the Additional file 1. Mean scores, representing the mean of the average of the two reviewer scores, were calculated for each category. Calculated means were as follows: total mean score 18.7 out of 32 (SD: 2.9, range 11–25.5); reporting sub-scale mean score 8.4 out of 11 (SD: 1.3, range 4.5–10.5); external validity sub-scale mean score 1.0 out of 3 (SD: 0.8, range 0–3); bias sub-scale mean score 4.3 out of 7 (SD: 0.5, range 3–5); confounding sub-scale mean score 2.5 out of 6 (SD: 0.8, range 0.5–4); power sub-scale mean score 4.7 out of 5 (SD: 0.9, range 1–5). The three RCTs that were critically appraised using version 2 of the Cochrane tool for assessing risk of bias in randomised trials [19] were found to range between having some concerns of risk of bias to low risk of bias [31, 55, 60].

## Discussion

To our knowledge, this is the first systematic literature review and meta-analysis to characterize depressive symptoms after stoma surgery and identify potential predictive factors. Depressive symptoms occur in almost half (43% across all timepoints) of stoma surgery patients, however mostly occur at a level of severity below the DSM-5 clinical threshold for major depressive disorder, based on pooled scores of validated depression measures (HADS, BDI, PHQ-9) and their associated severity criteria [3]. Given that this rate of depressive symptoms is significantly higher than the reported rates (under 20%) in the general population, both before and after COVID-19 [90], and also many inflammatory bowel disease [91] and colorectal cancer [92] populations presented in the peer-reviewed literature, it is likely that a proportion of this figure is a direct result of stoma surgery itself, however it is also likely that another proportion is as a result of having and managing a stoma in the short and long-term, and the psychosocial modifications that accompany this. Pooled scores for respective validated depression measures (HADS, BDI, and PHQ-9) across studies reporting those scores were below clinical thresholds for major depressive disorder according to severity criteria of the respective scores. Geographic region was significantly predictive of experiencing postoperative depressive symptoms, whereas age and sex were not. Odds of experiencing depressive symptoms were greater after stoma surgery versus before, and were less frequent in non-stoma surgical populations versus stoma surgical populations for studies conducting this comparison and using the validated HADS measure. However, of those experiencing depression after surgery, depressive symptoms were greater in non-stoma versus stoma populations. Ileostomy patients were less likely to experience postoperative depressive symptoms compared with colostomy patients, as were those with a permanent stoma versus a temporary stoma. Higher rates of depressive symptoms were reported after stoma surgery for patients with active Crohn’s disease versus those without, which may be considered surprising given that stomas are expected to improve quality of life in many of these cases, particularly in Crohn’s disease patients who fail to respond to medical therapy. The evidence for the association between time since surgery and depressive symptoms is mixed, with three studies reporting no association, a positive association, and a negative association respectively [24, 30, 79]. This may be considered surprising as many of the patient populations within these studies involved those with colorectal cancer, as there may be expected to be a period of post-traumatic growth (enduring positive psychological change as a result of adversity, trauma, or challenging life circumstances) [93] after undergoing life events such as stoma surgery, and it could be hypothesised that these patients may be experiencing depressive symptoms that vary depending on their response to their cancer, as opposed to the stoma itself. Methodological quality varied across the included studies as assessed by the Downs and Black and Cochrane RoB 2.0 checklists. According to Downs and Black checklist, the 65 NRSIs were of low to moderate methodological quality. According to Cochrane RoB2, the three RCTs ranged from low risk of bias to some concerns of bias.

The pathophysiology of depression comprises both psychosocial and biological components, and there is strong evidence of a causal link between environmental milieu and depressive symptoms [4]. Psychological adjustment before and after stoma surgery can be conceptualised within Engel’s biopsychosocial model [9] as an aggregation of stressors that patients experience and must manage: physical symptoms of their illness, diagnosis, the informed decision to have surgery, undergoing surgery, and then managing the resultant stoma bag and its associated implications. A considerable biological toll is exacted from these patients as they have been burdened with a significant chronic disease, and undergo major surgery to produce a stoma. The significant morbidity that usually follows stoma surgery is well established [94]. Most of the recent literature in this area relates to the psychosocial changes that occur after surgery [11, 12, 95, 96]. Patients likely undergo a form of grieving as they experience a loss of self-concept during psychological adjustment to their stoma [97]; losing elements of their independence, body-image, self-worth, hobbies, relationships, and intimacy. Despite historical advances in stoma appliances and stomal nursing care, the psychosocial adjustment required by patients is still considerable [98]. In addition to developing stoma care self-efficacy, learning to accept the necessity of the stoma bag and its care despite prominent social stigma and also maintaining interpersonal relationships may be challenging for stoma surgery patients [99]. Throughout the initial adjustment after surgery, strength of social supports may be as important as access to stoma care. Further, cost-effective methods of enhancing shared decision-making may improve patient adherence to management plans as with other forms of chronic disease [100].

Although there were insufficient data to be evaluated within this present study, the effect of socioeconomic status, city versus rural setting, urgency of stoma surgery, cultural differences, strength of familial and community supports, and participation in stoma support groups should be explored in future research for the benefit of stoma care. Given that psychological adjustment to stoma surgery encompasses a wide range of biological, psychological, and socioeconomic factors, it is likely that vulnerable populations will face inequalities in health outcomes [101]. The authors hypothesise that this may provide an explanation for the significant difference between geographical region in occurrence of depressive symptoms after stoma surgery that was found in the present study. Separate to patients who have undergone stoma surgery, variation at a country level in depressive symptoms has been well described in the prominent literature, with individual-level factors, socioeconomic and sociocultural inequality, and population characteristics being identified as having significant association with observed differences [102, 103]. Given the importance of patient demographics, and sociocultural and socioeconomic milieu, on the care and biopsychosocial outcomes of patients who have undergone stoma surgery, it is intuitive that depressive symptoms in this patient population would vary significantly according to geographical region when all of these associated factors also vary significantly according to geographical region. Of note within the present study’s results, although geographic region was found to be a factor that was significantly associated with depressive symptoms after stoma surgery, in direct comparisons between regions, only the Middle East / Africa versus Asia–Pacific was found to be significantly different with regards to depressive symptoms (differences in Europe versus Asia–Pacific and North America versus Asia–Pacific comparisons were not statistically significant). These findings could potentially reflect sociocultural differences and differences in approaches to the perioperative biopsychosocial care of patients undergoing stoma surgery between the two geographical regions. Going forward, these findings should be evaluated through robustly designed studies and findings integrated within evidence-based care that span multiple geographic regions and patient populations.

The greater incidence and severity of mood disorders following colostomy is likely attributable to a myriad of social and biological factors. From a biological perspective, two key concepts have emerged over the past decade which may contribute to the development of mood disorders: inflammation and the microbiome. Under homeostatic conditions, bidirectional communication exists between the resident microbiome and the gut-brain axis, with the microbiome releasing neuroactive molecules including short-chain fatty acids, neurotransmitters (e.g. GABA), hormones, and immune modulators. In addition, the microbiome synthesizes precursors for several neurotransmitters (e.g. L-tryptophan) [104]. These microbiome-derived molecules influence brain development, psychology, and in turn behaviour [105]. Insults which disrupt or alter the microbiome's composition (and hence the type of molecules released) have been associated with alterations in behaviour and the development of mood disorders in humans and animals [104]. Furthermore, patients with depression have shown gut microbiome dysbiosis [106]. Since surgical resection of the colon is a significant insult that unavoidably modifies the microbiome's composition (due to perioperative interventions [bowel preparation and antibiotics] and the surgery itself [resection, transplant, stoma formation]) [107, 108], and the current results demonstrate an increased prevalence of mood disorders following surgery, it is interesting to speculate whether microbiome modifications contribute to the development of mood disorders in these patients, and whether interventions aimed at modifying the microbiome may improve their condition.

Inflammatory processes are implicated in the pathophysiology of depression [1]. The bidirectional relationship between depression and inflammation has changed across evolutionary time, potentially explaining greater rates of depression in the more sanitary environments of modern societies [109]. About a quarter of patients with depression likely display evidence of low-grade inflammation, with over half of patients having mildly elevated C-Reactive Protein levels [110]. Stoma surgery is most frequently conducted in the treatment of colorectal cancer or inflammatory bowel disease. For both diseases, inflammation is key within the pathophysiology [111, 112]. While the psychosocial changes that occur in patients’ lives after stoma surgery are important to the considerable rates of postoperative depressive symptoms, it is possible that there is also a biological component of inflammation within the multifaceted pathogenesis [4]. Further research comparing depressive symptoms after surgery in patient populations with inflammatory diseases versus those without, is needed to clarify the role that inflammation plays in these patients’ postoperative psychological symptoms and whether non-steroidal anti-inflammatory drugs may have utility [113].

This study’s findings add to the growing evidence that mental health issues occur after stoma surgery, and echo the need for greater prioritisation within clinical care [11, 12, 95, 96]. Going forward, perioperative protocols should be modified to optimise psychological adjustment following significant surgery-induced changes in functional capacity or body image. Recent studies have indicated that increased access to psychological support may be beneficial for certain stoma surgery populations [114]. Along with preoperative workup of biological risks that could worsen surgical and anaesthetic outcomes, more rigorous psychological evaluation should be conducted before surgery to screen for individuals vulnerable to worse psychological outcomes. After surgery occurs, psychological evaluation must be undertaken in the immediate postoperative period so that longer-term adjustment can be optimised and evidence-based psychotherapy or pharmacotherapy implemented if appropriate. Given that most of the postoperative depressive symptoms characterised in this study did not reach the clinical threshold for major depressive disorder [3], a multidisciplinary approach should be undertaken early in this patient population to prevent progression past this threshold.

This study has several limitations that are inherent to studies of this kind. Both validated and non-validated measures of depressive symptoms were used within the included studies, and this may have added bias to subsequent findings. The included studies varied widely in their methodological quality, and in their study design ranging from qualitative studies to RCTs; both these factors increase the risk of bias within the conclusions. However, the large evidence base and rigorous adherence to internationally accepted reporting guidelines [13, 14] that we employed should improve the reliability of this study's findings. The diagnostic definition of major depressive disorder within the DSM-5 is multifaceted, reflecting the reality that depression can present in various ways [3]. Accordingly heterogeneity was found regarding the definition of depression in many of the included studies. To minimise any possible resultant bias, our review search strategies were designed in accordance with the DSM-5 definition of major depressive disorder, and emphasis was given to raw rates of depressive symptoms within stoma surgery populations as opposed to their severity.

## Conclusion

Depressive symptoms occur in almost half of stoma surgery patients, however mostly occur at a level of severity below the DSM-5 clinical threshold for major depressive disorder. Geographic region may be predictive of these postoperative depressive symptoms. Type of stoma, stoma permanency, and surgical pathology may influence the development of depressive symptoms after stoma surgery. Perioperative care may be enhanced by increased psychological evaluation to screen for vulnerable individuals in the preoperative period, and detect depressive symptoms in the postoperative period so that psychosocial adjustment can be optimised via appropriate psychotherapy. Future research should investigate depressive symptoms before versus after stoma surgery, and sub-analysed and controlled for different pathology indications for surgery.

## Supplementary Information

**Additional file 1: Appendix 1. **PRISMAChecklist. **Appendix 2. **Studies included at full-text review. **Supplementary Figure 1.** Funnel plot summarising publication bias for studies with data regarding effect of age on depressive symptoms after stoma surgery. **Supplementary Figure 2.** Funnel plot summarising publication bias for studies with data regarding mean age and standard deviations in studies with data regarding effect of age on depressive symptoms after stoma surgery. **Supplementary Figure 3.** Funnel plot summarising publication bias for studies with data regarding effect of sex on depressive symptoms after stoma surgery. **Supplementary Figure 4.** Forest plot summarising prevalence of male sex in studies with data regarding effect of sex on depressive symptoms after stoma surgery. **Supplementary Figure 5.** Funnel plot summarising publication bias for studies with data regarding prevalence of male sex and data regarding effect of sex on depressive symptoms after stoma surgery. **Supplementary Figure 6.** Forest plot showing prevalence of depressive symptoms after stoma surgery in studies reporting by region. **Supplementary Figure 7.** Funnel plot summarising publication bias for studies with data regarding effect of region on depressive symptoms after stoma surgery. **Supplementary Figure 8.** Forest plot summarising the odds of experiencing depressive symptoms before versus after stoma surgery. **Supplementary Figure 9.** Funnel plot summarising publication bias in studies reporting odds of experiencing depressive symptoms before versus after stoma surgery. **Supplementary Figure 10.** Forest plot summarising the odds of experiencing depressive symptoms after surgery in stoma versus non-stoma populations. **Supplementary Figure 11.** Funnel plot summarising publication bias in studies reporting data regarding the odds of experiencing depressive symptoms after surgery in stoma versus non-stoma populations. **Supplementary Figure 12.** Forest plot summarising mean differences in experiencing depressive symptoms after surgery in stoma versus non-stoma populations. **Supplementary Figure 13.** Funnel plot summarising publication bias in studies reporting data regarding mean differences in experiencing depressive symptoms after surgery in stoma versus non-stoma populations. **Supplementary Figure 14.** Forest plot summarising the mean differences in experiencing depressive symptoms after surgery in colostomy versus ileostomy patients. **Supplementary Figure 15.** Funnel plot summarising publication bias in studies reporting data regarding the mean differences in experiencing depressive symptoms after surgery in colostomy versus ileostomy patients. **Supplementary Table 1.** Risk of bias assessment using the Downs and Black Checklist for 65 non-randomised studies of interventions included in the present systematic review. **Supplementary Table 2.** Overall risk of bias assessment using the Cochrane RoB 2.0 checklist for three randomised controlled trials included in the present systematic review. 

## Data Availability

The datasets used and/or analysed during the current study available from the corresponding author on reasonable request.
