# Supplementary material for: Depression after stoma surgery: a systematic review and meta-analysis
Source: BMC Psychiatry. 2023 May 22;23:352. doi: 10.1186/s12888-023-04871-0 (PMC10201486; doi:10.1186/s12888-023-04871-0)
Supplement: Supplementary file 1 — Additional file 1: Appendix 1. PRISMAChecklist. Appendix 2. Studies included at full-text review. Supplementary Figure 1. Funnel plot summarising publication bias for studies with data regarding effect of age on depressive symptoms after stoma surgery. Supplementary Figure 2. Funnel plot summarising publication bias for studies with data regarding mean age and standard deviations in studies with data regarding effect of age on depressive symptoms after stoma surgery. Supplementary Figure 3. Funnel plot summarising publication bias for studies with data regarding effect of sex on depressive symptoms after stoma surgery. Supplementary Figure 4. Forest plot summarising prevalence of male sex in studies with data regarding effect of sex on depressive symptoms after stoma surgery. Supplementary Figure 5. Funnel plot summarising publication bias for studies with data regarding prevalence of male sex and data regarding effect of sex on depressive symptoms after stoma surgery. Supplementary Figure 6. Forest plot showing prevalence of depressive symptoms after stoma surgery in studies reporting by region. Supplementary Figure 7. Funnel plot summarising publication bias for studies with data regarding effect of region on depressive symptoms after stoma surgery. Supplementary Figure 8. Forest plot summarising the odds of experiencing depressive symptoms before versus after stoma surgery. Supplementary Figure 9. Funnel plot summarising publication bias in studies reporting odds of experiencing depressive symptoms before versus after stoma surgery. Supplementary Figure 10. Forest plot summarising the odds of experiencing depressive symptoms after surgery in stoma versus non-stoma populations. Supplementary Figure 11. Funnel plot summarising publication bias in studies reporting data regarding the odds of experiencing depressive symptoms after surgery in stoma versus non-stoma populations. Supplementary Figure 12. Forest plot summarising mean differences in experiencing depressiv [file 12888_2023_4871_MOESM1_ESM.docx]

**SUPPLEMENTARY APPENDIX**

**APPENDIX 1**

**PRISMA Checklist**

| **Section and Topic** | **Item #** | **Checklist item** | **Location where item is reported (page)** |
| --- | --- | --- | --- |
| **TITLE** | | |  |
| Title | 1 | Identify the report as a systematic review. | 1 |
| **ABSTRACT** | | |  |
| Abstract | 2 | See the PRISMA 2020 for Abstracts checklist. | 5 |
| **INTRODUCTION** | | |  |
| Rationale | 3 | Describe the rationale for the review in the context of existing knowledge. | 6 |
| Objectives | 4 | Provide an explicit statement of the objective(s) or question(s) the review addresses. | 6 |
| **METHODS** | | |  |
| Eligibility criteria | 5 | Specify the inclusion and exclusion criteria for the review and how studies were grouped for the syntheses. | 7 |
| Information sources | 6 | Specify all databases, registers, websites, organisations, reference lists and other sources searched or consulted to identify studies. Specify the date when each source was last searched or consulted. | 7 |
| Search strategy | 7 | Present the full search strategies for all databases, registers and websites, including any filters and limits used. | 7, appendix |
| Selection process | 8 | Specify the methods used to decide whether a study met the inclusion criteria of the review, including how many reviewers screened each record and each report retrieved, whether they worked independently, and if applicable, details of automation tools used in the process. | 6-8 |
| Data collection process | 9 | Specify the methods used to collect data from reports, including how many reviewers collected data from each report, whether they worked independently, any processes for obtaining or confirming data from study investigators, and if applicable, details of automation tools used in the process. | 7,8 |
| Data items | 10a | List and define all outcomes for which data were sought. Specify whether all results that were compatible with each outcome domain in each study were sought (e.g. for all measures, time points, analyses), and if not, the methods used to decide which results to collect. | 7,8 |
|  | 10b | List and define all other variables for which data were sought (e.g. participant and intervention characteristics, funding sources). Describe any assumptions made about any missing or unclear information. | 7,8 |
| Study risk of bias assessment | 11 | Specify the methods used to assess risk of bias in the included studies, including details of the tool(s) used, how many reviewers assessed each study and whether they worked independently, and if applicable, details of automation tools used in the process. | 8 |
| Effect measures | 12 | Specify for each outcome the effect measure(s) (e.g. risk ratio, mean difference) used in the synthesis or presentation of results. | 7,8 |
| Synthesis methods | 13a | Describe the processes used to decide which studies were eligible for each synthesis (e.g. tabulating the study intervention characteristics and comparing against the planned groups for each synthesis (item #5)). | 7,8 |
|  | 13b | Describe any methods required to prepare the data for presentation or synthesis, such as handling of missing summary statistics, or data conversions. | 7,8 |
|  | 13c | Describe any methods used to tabulate or visually display results of individual studies and syntheses. | 7,8 |
|  | 13d | Describe any methods used to synthesize results and provide a rationale for the choice(s). If meta-analysis was performed, describe the model(s), method(s) to identify the presence and extent of statistical heterogeneity, and software package(s) used. | 7,8 |
|  | 13e | Describe any methods used to explore possible causes of heterogeneity among study results (e.g. subgroup analysis, meta-regression). | 7,8 |
|  | 13f | Describe any sensitivity analyses conducted to assess robustness of the synthesized results. | 7,8 |
| Reporting bias assessment | 14 | Describe any methods used to assess risk of bias due to missing results in a synthesis (arising from reporting biases). | 7,8 |
| Certainty assessment | 15 | Describe any methods used to assess certainty (or confidence) in the body of evidence for an outcome. | 7,8 |
| **RESULTS** | | |  |
| Study selection | 16a | Describe the results of the search and selection process, from the number of records identified in the search to the number of studies included in the review, ideally using a flow diagram. | 8,9, figure 1 |
|  | 16b | Cite studies that might appear to meet the inclusion criteria, but which were excluded, and explain why they were excluded. | 8,9, figure 1, appendix |
| Study characteristics | 17 | Cite each included study and present its characteristics. | 8, 9, table 1 |
| Risk of bias in studies | 18 | Present assessments of risk of bias for each included study. | 8,9,14 |
| Results of individual studies | 19 | For all outcomes, present, for each study: (a) summary statistics for each group (where appropriate) and (b) an effect estimate and its precision (e.g. confidence/credible interval), ideally using structured tables or plots. | 8-14, table 1, 2, figures 1-4, appendix |
| Results of syntheses | 20a | For each synthesis, briefly summarise the characteristics and risk of bias among contributing studies. | 8-14, table 1, 2, figures 1-4, appendix |
|  | 20b | Present results of all statistical syntheses conducted. If meta-analysis was done, present for each the summary estimate and its precision (e.g. confidence/credible interval) and measures of statistical heterogeneity. If comparing groups, describe the direction of the effect. | 8-14, table 1, 2, figures 1-4, appendix |
|  | 20c | Present results of all investigations of possible causes of heterogeneity among study results. | 8-14, table 1, 2, figures 1-4, appendix |
|  | 20d | Present results of all sensitivity analyses conducted to assess the robustness of the synthesized results. | 8-14, table 1, 2, figures 1-4, appendix |
| Reporting biases | 21 | Present assessments of risk of bias due to missing results (arising from reporting biases) for each synthesis assessed. | 8-14, table 1, 2, figures 1-4, appendix |
| Certainty of evidence | 22 | Present assessments of certainty (or confidence) in the body of evidence for each outcome assessed. | 8-14, table 1, 2, figures 1-4, appendix |
| **DISCUSSION** | | |  |
| Discussion | 23a | Provide a general interpretation of the results in the context of other evidence. | 14-18 |
|  | 23b | Discuss any limitations of the evidence included in the review. | 17,18 |
|  | 23c | Discuss any limitations of the review processes used. | 17,18 |
|  | 23d | Discuss implications of the results for practice, policy, and future research. | 14-18 |
| **OTHER INFORMATION** | | |  |
| Registration and protocol | 24a | Provide registration information for the review, including register name and registration number, or state that the review was not registered. | 6 |
|  | 24b | Indicate where the review protocol can be accessed, or state that a protocol was not prepared. | 6 |
|  | 24c | Describe and explain any amendments to information provided at registration or in the protocol. | 6 |
| Support | 25 | Describe sources of financial or non-financial support for the review, and the role of the funders or sponsors in the review. | 2 |
| Competing interests | 26 | Declare any competing interests of review authors. | 2 |
| Availability of data, code and other materials | 27 | Report which of the following are publicly available and where they can be found: template data collection forms; data extracted from included studies; data used for all analyses; analytic code; any other materials used in the review. | 2 |

**Search Strategies**

**Updated searches: 19 June 2021 – 6 March 2023**

**PubMed (incorporating MEDLINE)**

(stoma OR colostom* OR ileostom*) AND (depress* OR interest OR anhedonia)

970 results

**Embase***

(stoma OR colostomy OR ileostomy) AND (depression OR interest OR anhedonia)

266 results

**CINAHL***

(stoma OR colostom* OR ileostom*) AND (depress* OR interest OR anhedonia)

18 results

**Cochrane Library – Trials**

(stoma OR colostom* OR ileostom*) AND (depress* OR interest OR anhedonia)

24 results

*date limit from 2021 to 2023

**Initial searches: Inception – 19 June 2021**

**PubMed (incorporating MEDLINE)**

(stoma OR colostom* OR ileostom*) AND (depress* OR interest OR anhedonia)

1,941 results

**Embase**

(stoma OR colostomy OR ileostomy) AND (depression OR interest OR anhedonia)

1,136 results

**CINAHL**

(stoma OR colostom* OR ileostom*) AND (depress* OR interest OR anhedonia)

2,583 results

**Cochrane Library – Trials**

(stoma OR colostom* OR ileostom*) AND (depress* OR interest OR anhedonia)

82 results

**APPENDIX 2**

**Studies included at full-text review**

1. Abdalla, Maisa I., et al. "The impact of ostomy on quality of life and functional status of Crohn's disease patients." Inflammatory bowel diseases 22.11 (2016): 2658-2664.
2. Ananthakrishnan, Ashwin N., et al. "Similar risk of depression and anxiety following surgery or hospitalization for Crohn’s disease and ulcerative colitis." The American journal of gastroenterology 108.4 (2013): 594.
3. Anaraki, Fakhryalsadat, et al. "Clinical profile and post-operative lifestyle changes in cancer and non-cancer patients with ostomy." Gastroenterology and Hepatology from bed to bench 5.Suppl 1 (2012): S26.
4. Anaraki, Fakhrialsadat, et al. "Quality of life outcomes in patients living with stoma." Indian journal of palliative care 18.3 (2012): 176.
5. Armbruster, Shannon D., et al. "Prospective assessment of patient-reported outcomes in gynecologic cancer patients before and after pelvic exenteration." Gynecologic oncology 149.3 (2018): 484-490.
6. Bahayi, Kader, et al. "Depression, anxiety, sexual dysfunction and quality of life in patients with ileostomy or colostomy." Turk J Colorectal Dis 28.2 (2018): 69-75.
7. Barisic, G., et al. "Function after intersphincteric resection for low rectal cancer and its influence on quality of life." Colorectal Disease 13.6 (2011): 638-643.
8. Bau, M. O., et al. "The Malone antegrade colonic enema isolated or associated with urological incontinence procedures: evaluation from patient point of view." The Journal of urology 165.6 Part 2 (2001): 2399-2403.
9. Blackwell, J., et al. "Stoma formation in Crohn’s Disease and the likelihood of antidepressant use: a population-based cohort study." Clinical Gastroenterology and Hepatology (2020).
10. Bossema, Ercolie R., et al. "The relation between illness cognitions and quality of life in people with and without a stoma following rectal cancer treatment." Psycho‐oncology 20.4 (2011): 428-434.
11. Bullen, Tracey L., et al. "Body image as a predictor of psychopathology in surgical patients with colorectal disease." Journal of psychosomatic research 73.6 (2012): 459-463.
12. Chaudhri, Sanjay, et al. "Preoperative intensive, community-based vs. traditional stoma education: a randomized, controlled trial." Diseases of the colon & rectum 48.3 (2005): 504-509.
13. Chen, Allen M., et al. "Depression among long-term survivors of head and neck cancer treated with radiation therapy." JAMA Otolaryngology–head & Neck Surgery 139.9 (2013): 885-889.
14. Coggrave, M. J., et al. "The impact of stoma for bowel management after spinal cord injury." Spinal Cord 50.11 (2012): 848-852.
15. Colquhoun, Patrick, et al. "Is the quality of life better in patients with colostomy than patients with fecal incontience?." World journal of surgery 30.10 (2006): 1925-1928.
16. Cotrim, Hortense, and Graça Pereira. "Impact of colorectal cancer on patient and family: implications for care." European Journal of Oncology Nursing 12.3 (2008): 217-226.
17. Davidson, Fiona. "Quality of life, wellbeing and care needs of Irish ostomates." British journal of nursing 25.17 (2016): S4-S12.
18. Davis, Deena, Lakshmi Ramamoorthy, and Biju Pottakkat. "Impact of stoma on lifestyle and health-related quality of life in patients living with stoma: A cross-sectional study." Journal of Education and Health Promotion 9 (2020).
19. Geng, Zhaohui, et al. "Quality of life in Chinese persons living with an ostomy." Journal of Wound, Ostomy and Continence Nursing 44.3 (2017): 249-256.
20. González, Elisabeth, et al. "Self-reported wellbeing and body image after abdominoperineal excision for rectal cancer." International journal of colorectal disease 31.10 (2016): 1711-1717.
21. Grant, Marcia, et al. "Gender differences in quality of life among long-term colorectal cancer survivors with ostomies." Oncology nursing forum. Vol. 38. No. 5. NIH Public Access, 2011.
22. Holzer, Brigitte, et al. "Do geographic and educational factors influence the quality of life in rectal cancer patients with a permanent colostomy?." Diseases of the colon & rectum 48.12 (2005): 2209-2216.
23. Hong, Kyung Sook, et al. "Psychological attitude to self-appraisal of stoma patients: prospective observation of stoma duration effect to self-appraisal." Annals of surgical treatment and research 86.3 (2014): 152-160.
24. Hornbrook, Mark C., et al. "Complications among colorectal cancer survivors: SF-6D preference-weighted quality of life scores." Medical care 49.3 (2011): 321.
25. Iqbal, Fareed, et al. "Patient-reported outcome after ostomy surgery for chronic constipation." Journal of Wound Ostomy & Continence Nursing 45.4 (2018): 319-325.
26. Jayarajah, Umesh, and Dharmabandhu Nandadeva Samarasekera. "Psychological adaptation to alteration of body image among stoma patients: a descriptive study." Indian journal of psychological medicine 39.1 (2017): 63-68.
27. Jayarajah, Umesh, and Dharmabandhu N. Samarasekera. "A cross-sectional study of quality of life in a cohort of enteral ostomy patients presenting to a tertiary care hospital in a developing country in South Asia." BMC research notes 10.1 (2017): 1-8.
28. Jin, Ying, et al. "Psychosocial behaviour reactions, psychosocial needs, anxiety and depression among patients with rectal cancer before and after colostomy surgery: A longitudinal study." Journal of clinical nursing 28.19-20 (2019): 3547-3555.
29. Karakayali, Feza Yarbug, et al. "The Outcomes of Ultralow Anterior Resection or an Abdominoperineal Pull-Through Resection and Coloanal Anastomosis for Radiation-Induced Recto-Vaginal Fistula Patients." Journal of Gastrointestinal Surgery 20.5 (2016): 994-1001.
30. Keltikangas-Järvinen, Liisa, and Eija-Liisa Loven. "Stability of personality dimensions related to cancer and colitis ulcerosa: preliminary report." Psychological reports 52.3 (1983): 961-962.
31. Keltikangas-Järvinen, Liisa, Eija Loven, and Carl Möller. "Psychic factors determining the long-term adaptation of colostomy and ileostomy patients." Psychotherapy and psychosomatics 41.3 (1984): 153-159.
32. Ketterer, Sarah N., Michael J. Leach, and Caitlin Fraser. "Factors Associated with Quality of Life Among People Living With a Stoma in Nonmetropolitan Areas." Nursing Research (2021).
33. Knowles, S. R., S. I. Cook, and D. Tribbick. "Relationship between health status, illness perceptions, coping strategies and psychological morbidity: a preliminary study with IBD stoma patients." Journal of Crohn's and Colitis 7.10 (2013): e471-e478.
34. Knowles, Simon R., et al. "Psychological well-being and quality of life in Crohn's disease patients with an ostomy: a preliminary investigation." Journal of Wound Ostomy & Continence Nursing 40.6 (2013): 623-629.
35. Knowles, Simon R., et al. "Exploration of health status, illness perceptions, coping strategies, and psychological morbidity in stoma patients." Journal of Wound Ostomy & Continence Nursing 41.6 (2014): 573-580.
36. Krouse, Robert S., et al. "Health-related quality of life among long-term rectal cancer survivors with an ostomy: manifestations by sex." Journal of Clinical Oncology 27.28 (2009): 4664.
37. Krouse, Robert S., et al. "A chronic care ostomy self‐management program for cancer survivors." Psycho‐Oncology 25.5 (2016): 574-581.
38. Lamb, Karen A., and Jen Lodge. "Managing symptoms associated with the redundant distal colon and rectum: a pilot scoping exercise." Gastrointestinal Nursing 17.1 (2019): 20-24.
39. Lim, Siew Hoon, et al. "Pilot trial of a STOMA psychosocial intervention programme for colorectal cancer patients with stomas." Journal of advanced nursing 75.6 (2019): 1338-1346
40. LIU, Y., and LI NI. "Construction and Application of Remote Continuing Care Model for Colorectal Cancer Patients in the Internet Era." Indian Journal of Pharmaceutical Sciences (2021): 177-181.
41. Lowe, Bethany Grace, Eman Alsaleh, and Holly Blake. "Assessing physical activity levels in people living with a stoma." Nursing standard (Royal College of Nursing (Great Britain): 1987) 35.1 (2019): 70-77.
42. MacDonald, L. D., and H. R. Anderson. "The health of rectal cancer patients in the community." European journal of surgical oncology 11.3 (1985): 235-241.
43. Mohamed, Nihal E., et al. "Dealing With the Unthinkable: Bladder and Colorectal Cancer Patients’ and Informal Caregivers’ Unmet Needs and Challenges in Life After Ostomies." Seminars in oncology nursing. WB Saunders, 2021.
44. Mols, Floortje, et al. "Living with the physical and mental consequences of an ostomy: a study among 1–10‐year rectal cancer survivors from the population‐based PROFILES registry." Psycho‐oncology 23.9 (2014): 998-1004.
45. Norton, Christine, Jennie Burch, and Michael A. Kamm. "Patients’ views of a colostomy for fecal incontinence." Diseases of the colon & rectum 48.5 (2005): 1062-1069.
46. Park, Seungmi, In Sun Jang, and Yeon S. Kim. "Risks for depression among ostomates in South Korea." Japan Journal of Nursing Science 15.3 (2018): 203-209.
47. Portier, Guillaume, et al. "Use of Malone antegrade continence enema in patients with perineal colostomy after rectal resection." Diseases of the colon & rectum 48.3 (2005): 499-503.
48. Powell‐Chandler, A., et al. "Psychological sequelae of colonic resections." Colorectal Disease 22.8 (2020): 945-951.
49. Rafiei, Hossein, et al. "The prevalence of psychological problems among ostomy patients: A cross-sectional study from Iran." Gastrointestinal Nursing 15.2 (2017): 39-44.
50. Rafiei, Hossein, et al. "The relationship between psychological health and spiritual wellbeing in Iranian stoma patients." Gastrointestinal Nursing 17.Sup5 (2019): S18-S22.
51. Ramer, Lois. "Self-image changes with time in the cancer patient with a colostomy after operation." Journal of ET nursing: official publication, International Association for Enterostomal Therapy 19.6 (1992): 195-203.
52. Reese, J. B., et al. "Gastrointestinal ostomies and sexual outcomes: a comparison of colorectal cancer patients by ostomy status." Supportive care in cancer 22.2 (2014): 461-468.
53. Repić, Gordana, et al. "Psychological and spiritual well-being aspects of the quality of life in colostomy patients." Vojnosanitetski pregled 75.6 (2018): 611-617.
54. Richbourg, Leanne, Joshua M. Thorpe, and Carla Gene Rapp. "Difficulties experienced by the ostomate after hospital discharge." Journal of Wound Ostomy & Continence Nursing 34.1 (2007): 70-79.
55. Sceats, L. A., et al. "Surgery, stomas, and anxiety and depression in inflammatory bowel disease: a retrospective cohort analysis of privately insured patients." Colorectal Disease 22.5 (2020): 544-553.
56. Sharpe, Louise, Deepa Patel, and Stephen Clarke. "The relationship between body image disturbance and distress in colorectal cancer patients with and without stomas." Journal of psychosomatic research 70.5 (2011): 395-402.
57. Song, Lili, et al. "Body image mediates the effect of stoma status on psychological distress and quality of life in patients with colorectal cancer." Psycho‐Oncology 29.4 (2020): 796-802.
58. Ssewanyana, Yasin, et al. "Quality of life of adult individuals with intestinal stomas in Uganda: a cross sectional study." African Health Sciences 21.1 (2021): 427-36.
59. Thomas, Chris, Felicity Madden, and Derek Jehu. "Psychological effects of stomas—I. Psychosocial morbidity one year after surgery." Journal of psychosomatic research 31.3 (1987): 311-316.
60. Wang, Peng, et al. "Extraperitoneal sigmoidostomy: a surgical approach with less complications and better functions for abdominoperineal resection of rectal cancer." International journal of colorectal disease 33.1 (2018): 41-46.
61. Williams, N. S., and D. Johnston. "The quality of life after rectal excision for low rectal cancer." Journal of British Surgery 70.8 (1983): 460-462.
62. Wirsching, Michael, H. U. Drüner, and G. Herrmann. "Results of psychosocial adjustment to long-term colostomy." Psychotherapy and psychosomatics 26.5 (1975): 245-256.
63. Koç, M. A., Akyol, C., Gökmen, D., Aydın, D., Erkek, B. A., & Kuzu, M. A. (2022). Effect of prehabilitation on stoma self-care, anxiety, depression and quality of life in stoma patients: a randomized controlled trial. *Diseases of the Colon & Rectum*.
64. Lemiński, A., Kaczmarek, K., Bańcarz, A., Zakrzewska, A., Małkiewicz, B., & Słojewski, M. (2021). Educational and psychological support combined with minimally invasive surgical technique reduces perioperative depression and anxiety in patients with bladder cancer undergoing radical cystectomy. *International Journal of Environmental Research and Public Health*, *18*(24), 13071.
65. Rud, C. L., Baunwall, S. M., Bager, P., Dahlerup, J. F., Wilkens, T. L., Tøttrup, A., ... & Hvas, C. L. (2022). Patient-reported outcomes and health-related quality of life in people living with ileostomies: a population-based, cross-sectional study. *Diseases of the Colon and Rectum*, *65*(8), 1042.
66. Shrestha, S., Siwakoti, S., Shakya, U., Shakya, R., & Khadka, S. (2022). Quality of Life, Anxiety and Depression among Clients with Ostomy Attending Selected Stoma Clinics. *Journal of Nepal Health Research Council*, *20*(02), 383-391.
67. Sivero, L., Bottone, M., Siciliano, S., Volpe, S., Maione, R., Chini, A., ... & Sivero, S. (2022). Post-operative oncological and psychological evaluation of patients with colostomy for colorectal cancer. *Annali italiani di chirurgia*, *93*(4), 435-438.
68. Zewude, W. C., Derese, T., Suga, Y., & Teklewold, B. (2021). Quality of life in patients living with stoma. *Ethiopian Journal of Health Sciences*, *31*(5).

**Studies excluded at full-text review**

**Wrong population**

1. Abu-Helalah, Munir Ahmad, et al. "Quality of life and psychological well-being of colorectal cancer survivors in Jordan." Asian Pacific Journal of Cancer Prevention 15.18 (2014): 7653-7664.
2. Ackerstaff, Annemieke H., et al. "Improvements in respiratory and psychosocial functioning following total laryngectomy by the use of a heat and moisture exchanger." Annals of Otology, Rhinology & Laryngology 102.11 (1993): 878-883.
3. Ahroni, Jessie H., Kevin F. Montgomery, and Brad M. Watkins. "Laparoscopic adjustable gastric banding: weight loss, co-morbidities, medication usage and quality of life at one year." Obesity surgery 15.5 (2005): 641-647.
4. Atallah, R., et al. "Safety, prosthesis wearing time and health-related quality of life of lower extremity bone-anchored prostheses using a press-fit titanium osseointegration implant: A prospective one-year follow-up cohort study." PloS one 15.3 (2020): e0230027.
5. Attarabeen, Omar F., et al. "Colon cancer worry in Appalachia." Journal of community health 43.1 (2018): 79-88.
6. Bourdais, Rémi, et al. "Pulse-dose-rate interstitial brachytherapy in anal squamous cell carcinoma: clinical outcomes and patients’ health quality perception." Journal of contemporary brachytherapy 13.3 (2021): 263.
7. Brandon, Tanya. "A portable, disposable system for negative-pressure wound therapy." British Journal of Nursing 24.2 (2015): 98-106.
8. Chang, Florence CF, et al. "Intraduodenal levodopa-carbidopa intestinal gel infusion improves both motor performance and quality of life in advanced Parkinson’s disease." Journal of Clinical Neuroscience 25 (2016): 41-45.
9. Chiloiro, Giuditta, et al. "Could the conservative approach be considered safe in the treatment of locally advanced rectal cancer in case of a clinical near-complete or complete response? A retrospective analysis." Clinical and translational radiation oncology 28 (2021): 1-9.
10. El-Gazzaz, G., et al. "Overlapping sphincter repair: does age matter?." Diseases of the colon & rectum 55.3 (2012): 256-261.
11. Fischer, J., et al. “Prophylactic mesh augmentation (PMA) in a high risk patient: A video case study”. Hernia 21 (1): S123
12. Fowkes, Lucy, et al. "Laparoscopic emergency and elective surgery for ulcerative colitis." Colorectal disease 10.4 (2008): 373-378.
13. Goudarzi, Zahra, et al. "The effect of educational program on stress, anxiety and depression of the mothers of neonates having colostomy." The Journal of Maternal-Fetal & Neonatal Medicine 29.23 (2016): 3902-3905.
14. Grundmann, R., S. Said, and S. Krinke. "Quality of life after rectal resection or extirpation. A comparison using different measurement parameters." Deutsche medizinische Wochenschrift (1946) 114.12 (1989): 453-457.
15. Hoeflok, Jo, et al. "Health-related quality of life in community-dwelling persons living with enterocutaneous fistulas." Journal of Wound Ostomy & Continence Nursing 42.6 (2015): 607-613.
16. Husson, Olga, et al. "The role of personality in the course of health-related quality of life and disease-specific health status among colorectal cancer survivors: a prospective population-based study from the PROFILES registry." Acta Oncologica 54.5 (2015): 669-677.
17. Kappelman, Michael D., et al. "Evaluation of the patient-reported outcomes measurement information system in a large cohort of patients with inflammatory bowel diseases." Clinical Gastroenterology and Hepatology 12.8 (2014): 1315-1323.
18. Keng, Christine JS, et al. "Home to stay: an integrated monitoring system using a mobile app to support patients at home following colorectal surgery." Journal of Patient Experience (2020): 2374373520904194.
19. Kornmann, Verena NN, et al. "Quality of life after a low anterior resection for rectal cancer in elderly patients." Annals of coloproctology 32.1 (2016): 27.
20. Lloyd, Shane, et al. "Mental health disorders are more common in colorectal cancer survivors and associated with decreased overall survival." American journal of clinical oncology 42.4 (2019): 355-362.
21. Creamer, Felicity, et al. "A European snapshot of psychosocial characteristics and patients’ perspectives of faecal incontinence—do they correlate with current scoring systems?." International journal of colorectal disease 36.6 (2021): 1175-1180.
22. Mahadev, Srihari, et al. "Self‐reported depressive symptoms and suicidal feelings in perianal Crohn’s disease." Colorectal Disease 14.3 (2012): 331-335.
23. Marks, John H., et al. "Quality of life and functional outcome after transanal abdominal transanal proctectomy for low rectal cancer." Diseases of the colon and rectum 60.3 (2017): 258.
24. Mielcarek, Piotr, Katarzyna Nowicka-Sauer, and Joanna Kozaka. "Anxiety and depression in patients with advanced ovarian cancer: a prospective study." Journal of Psychosomatic Obstetrics & Gynecology 37.2 (2016): 57-67.
25. Milbury, Kathrin, et al. "The association between psychosocial and medical factors with long-term sexual dysfunction after treatment for colorectal cancer." Supportive care in cancer 21.3 (2013): 793-802.
26. Montemurro, Severino, et al. "Sphincter-saving proctectomy for rectal cancer with NO COIL® transanal tube and without ostoma. Clinical outcomes, cost effectiveness and quality of life in the elderly." Minerva chirurgica 74.1 (2018): 19-25.
27. Nijboer, Chris, et al. "Determinants of caregiving experiences and mental health of partners of cancer patients." Cancer 86.4 (1999): 577-588.
28. Nordin, Karin, et al. "Health-related quality of life and psychological distress in a population-based sample of Swedish patients with inflammatory bowel disease." Scandinavian journal of gastroenterology 37.4 (2002): 450-457.
29. O’Toole, Aoibhlinn, et al. "Sexual dysfunction in men with inflammatory bowel disease: a new IBD-specific scale." Inflammatory bowel diseases 24.2 (2018): 310-316.
30. Panara, A. J., et al. "The incidence and risk factors for developing depression after being diagnosed with inflammatory bowel disease: a cohort study." Alimentary pharmacology & therapeutics 39.8 (2014): 802-810.
31. Pither, C., et al. "Psychiatric disorders in patients undergoing intestinal transplantation." Transplantation proceedings. Vol. 46. No. 6. Elsevier, 2014.
32. Qaderi, Seyed M., et al. "Follow‐up practice and healthcare utilisation of colorectal cancer survivors." European Journal of Cancer Care (2021): e13472.
33. Redelmeier, Donald A., and Daniel N. Heller. "Time preference in medical decision making and cost-effectiveness analysis." Medical Decision Making 13.3 (1993): 212-217.
34. Reese, Jennifer Barsky, Elizabeth Handorf, and Jennifer A. Haythornthwaite. "Sexual quality of life, body image distress, and psychosocial outcomes in colorectal cancer: a longitudinal study." Supportive Care in Cancer 26.10 (2018): 3431-3440.
35. Russell, Lahiru, et al. "Psychological distress, quality of life, symptoms and unmet needs of colorectal cancer survivors near the end of treatment." Journal of cancer survivorship 9.3 (2015): 462-470.
36. Sharma, A., et al. "Predictors of early postoperative quality of life after elective resection for colorectal cancer." Annals of Surgical Oncology 14.12 (2007): 3435-3442.
37. Snijders, H. S., et al. "Preoperative risk information and patient involvement in surgical treatment for rectal and sigmoid cancer." Colorectal Disease 16.2 (2014): O43-O49.
38. Stelzner, Matthias, J. Duncan Phillips, and Eric W. Fonkalsrud. "Acute ileus from steroid withdrawal simulating intestinal obstruction after surgery for ulcerative colitis." Archives of Surgery 125.7 (1990): 914-917.
39. Szigethy, Eva M., et al. "Depression subtypes in pediatric inflammatory bowel disease." Journal of pediatric gastroenterology and nutrition 58.5 (2014): 574.
40. Tajti Jr, János, et al. "Effect of laparoscopic surgery on quality of life in ulcerative colitis." Journal of Laparoendoscopic & Advanced Surgical Techniques 28.7 (2018): 833-838.
41. Valsdottir, Elsa B., et al. "Quality of life and fecal incontinence after transanal endoscopic microsurgery for benign and malignant rectal lesions." Surgical endoscopy 28.1 (2014): 193-202.
42. Wexner, Steven D., et al. "Long-term efficacy of dynamic graciloplasty for fecal incontinence." Diseases of the colon & rectum 45.6 (2002): 809-818.
43. Williet, Nicolas, et al. "Patient-reported outcomes in a French nationwide survey of inflammatory bowel disease patients." Journal of Crohn's and Colitis 11.2 (2017): 165-174.
44. Zutshi, M., et al. "Female bowel function: the real story." Diseases of the colon & rectum 50.3 (2007): 351-358.
45. Hashash, Jana G., et al. "Predictors of suicidal ideation among IBD outpatients." Journal of clinical gastroenterology 53.1 (2019): e41-e45.
46. Thomas, Chris, Felicity Madden, and Derek Jehu. "Psychological effects of stomas—II. factors influencing outcome." Journal of psychosomatic research 31.3 (1987): 317-323.
47. Ri, H., Kang, H., Xu, Z., Kim, K., Ren, Y., Gong, Z., & Chen, X. (2022). The risk factors of low anterior resection syndrome after colorectal cancer surgery: A retrospective study of 566 patients in a single institution in China. *Frontiers in Surgery*, 1263.

**Wrong outcomes**

1. Allin, Benjamin Saul Raywood, et al. "Outcomes at five to eight years of age for children with Hirschsprung’s disease." Archives of Disease in Childhood 106.5 (2021): 484-490.
2. Almutairi, K. M., et al. "A cross-sectional assessment of quality of life of colorectal cancer patients in Saudi Arabia." Clinical and Translational Oncology 18.2 (2016): 144-152.
3. Bulkley, Joanna, et al. "Spiritual well‐being in long‐term colorectal cancer survivors with ostomies." Psycho‐Oncology 22.11 (2013): 2513-2521.
4. Capolupo, Gabriella Teresa, et al. "Transanal proctocolectomy and ileal pouch-anal anastomosis (TaIPAA) for ulcerative colitis: medium term functional outcomes in a single centre." BMC surgery 21.1 (2021): 1-7.
5. Cheung, Yuk Lung, Alexander Molassiotis, and Anne M. Chang. "The effect of progressive muscle relaxation training on anxiety and quality of life after stoma surgery in colorectal cancer patients." Psycho‐Oncology: Journal of the Psychological, Social and Behavioral Dimensions of Cancer 12.3 (2003): 254-266.
6. Cheung, Y. L., A. Molassiotis, and A. M. Chang. "A pilot study on the effect of progressive muscle relaxation training of patients after stoma surgery." European Journal of Cancer Care 10.2 (2001): 107-114.
7. Collado-Boira, Eladio J., et al. "Self-Care and Health-Related Quality of Life in Patients with Drainage Enterostomy: A Multicenter, Cross Sectional Study." International Journal of Environmental Research and Public Health 18.5 (2021): 2443.
8. Damphousse, M., F. Beuret-Blanquart, and P. Denis. "Assessment of anorectal disorders with paraplegia." Annales de readaptation et de medecine physique: revue scientifique de la Societe francaise de reeducation fonctionnelle de readaptation et de medecine physique. Vol. 48. No. 5. 2005.
9. Danielsen, Anne Kjaergaard, and Jacob Rosenberg. "Health related quality of life may increase when patients with a stoma attend patient education–a case-control study." PLoS One 9.3 (2014): e90354.
10. SOUZA, José Luís da Costa Alves de, et al. "Health-related quality of life assessment in patients with rectal cancer treated with curative intent." Arquivos de gastroenterologia 55.2 (2018): 154-159.
11. Domislovic, Viktor, et al. "Prevalence, predictors and age‐related sexual and erectile dysfunction in patients with inflammatory bowel disease: a tertiary center experience." International Journal of Clinical Practice (2021): e14486.
12. Du, Xixi, et al. "The correlation between intimate relationship, self-disclosure, and adaptability among colorectal cancer enterostomy patients." Medicine 100.19 (2021).
13. Dulskas, Audrius, et al. "Quality of Life and Bowel Function Following Early Closure of a Temporary Ileostomy in Patients with Rectal Cancer: A Report from a Single-Center Randomized Controlled Trial." Journal of clinical medicine 10.4 (2021): 768.
14. Gautam, Sital, and Anju Poudel. "Effect of gender on psychosocial adjustment of colorectal cancer survivors with ostomy." Journal of gastrointestinal oncology 7.6 (2016): 938.
15. Goldstine, Jimena, et al. "Factors influencing health-related quality of life of those in the Netherlands living with an ostomy." British Journal of Nursing 28.22 (2019): S10-S17.
16. Grant, Madeleine. "Creative arts therapy in stoma care: workshops exploring grief, body image and sexual intimacy." Gastrointestinal Nursing 17.2 (2019): 24-29.
17. Hassine, Asma Ben, et al. "Quality of life in men after total cystoprostatectomy: Perceptions of Tunisian patients." Canadian Oncology Nursing Journal 29.4 (2019): 226.
18. Herrle, Florian, et al. "Quality of life and timing of stoma closure in patients with rectal cancer undergoing low anterior resection with diverting stoma: a multicenter longitudinal observational study." Diseases of the Colon & Rectum 59.4 (2016): 281-290.
19. Hubbard, Gill, et al. "A physical activity intervention to improve the quality of life of patients with a stoma: a feasibility study." Pilot and feasibility studies 6.1 (2020): 1-15.
20. Indrebø, Kirsten Lerum, et al. "Psychometric Properties of New Subscales of the Ostomy Adjustment Scale: A Cross-Sectional Study." Patient Related Outcome Measures 12 (2021): 65.
21. Jansen, Femke, et al. "A mixed-method study on the generic and ostomy-specific quality of life of cancer and non-cancer ostomy patients." Supportive Care in Cancer 23.6 (2015): 1689-1697.
22. Kerr, Jacqueline. et al "Doctor-patient communication." Diseases of the colon & rectum 46.8 (2003): 1038-1046.
23. Koedam, T. W. A., et al. "Transanal total mesorectal excision (TaTME) for rectal cancer: effects on patient-reported quality of life and functional outcome." Techniques in coloproctology 21.1 (2017): 25-33.
24. Konjevoda, Vesna, et al. "City of Hope Quality of Life-Ostomy Questionnaire Validity and Reliability Assessment on a Croatian Sample." International journal of environmental research and public health 17.3 (2020): 768.
25. Lam, Wendy WT, et al. "A longitudinal study of supportive care needs among Chinese patients awaiting colorectal cancer surgery." Psycho‐Oncology 25.5 (2016): 496-505.
26. Indrebø, Kirsten Lerum, John Roger Andersen, and Gerd Karin Natvig. "The Ostomy Adjustment Scale: translation into Norwegian language with validation and reliability testing." Journal of Wound Ostomy & Continence Nursing 41.4 (2014): 357-364.
27. Lim, Siew Hoon, et al. "A qualitative evaluation of the STOMA psychosocial intervention programme for colorectal cancer patients with stoma." Journal of advanced nursing 75.1 (2019): 108-118.
28. Lin, Jin-Bo, et al. "Validation of the chinese version of the EORTC QLQ-CR29 in patients with colorectal cancer." World journal of gastroenterology 23.10 (2017): 1891.
29. Laforest, A., et al. "Functional disorders after rectal cancer resection: does a rehabilitation programme improve anal continence and quality of life?." Colorectal Disease 14.10 (2012): 1231-1237.
30. Mayadevi, L., et al. "The city of hope quality of life stoma questionnaire: Malayalam translation and validation." Indian journal of palliative care 25.4 (2019): 556.
31. Maydick, Diane. "A Descriptive Study Assessing Quality of Life for Adults With a Permanent Ostomy and the Influence of Preoperative Stoma Site Marking." Ostomy/wound management 62.5 (2016): 14-24.
32. Mrak, K., et al. "Long‐term quality of life in pouch patients compared with stoma patients following rectal cancer surgery." Colorectal Disease 13.12 (2011): e403-e410.
33. Park, Seung-Mi, and Keum-Soon Kim. "Model construction of sexual satisfaction in patients with a colostomy." Journal of Korean Academy of Nursing 39.4 (2009): 539-548.
34. Patel, Frost, Bearn, Allan. `Canoe closure' of loop ileostomy gives improved cosmesis compared with conventional closure. Colorectal Dis. 1999 May;1(3):155-7. doi: 10.1046/j.1463-1318.1999.00024.x. PMID: 23577763.
35. L Perry-Woodford, Zarah. "Quality of life following ileoanal pouch failure." British Journal of Nursing 22.Sup11 (2013): S23-S28.
36. Planellas, Pere, et al. "Randomized clinical trial comparing side to end vs end to end techniques for colorectal anastomosis." International Journal of Surgery 83 (2020): 220-229.
37. Plata, Katarzyna, and Włodzimierz Majewski. "Quality of life of stoma patients after colorectal surgery and possibilities of its improvement." Annales Academiae Medicae Stetinensis. Vol. 54. No. 2. 2008.
38. Ratjen, Ilka, et al. "Health-related quality of life in long-term survivors of colorectal cancer and its association with all-cause mortality: a German cohort study." BMC cancer 18.1 (2018): 1-15.
39. Sakaguchi, Tatsuma, et al. "Postoperative complications of umbilical loop colostomy for anorectal malformations in neonates compared with the conventional abdominal stoma: a non-randomized study." World Journal of Pediatric Surgery 2.1 (2019).
40. Schaube, J., P. Scharf, and R. Herz. "The quality of life after extirpation of the rectum for carcinoma." Deutsche medizinische Wochenschrift (1946) 121.6 (1996): 153-7.
41. Silva, Karine de Almeida, et al. "Time after ostomy surgery and type of treatment are associated with quality of life changes in colorectal cancer patients with colostomy." PloS one 15.12 (2020): e0239201.
42. Silva, Michael Anthony, Geethani Ratnayake, and Kemal I. Deen. "Quality of life of stoma patients: temporary ileostomy versus colostomy." World journal of surgery 27.4 (2003): 421-424.
43. Sinha, Aditi, et al. "Quality of life of ostomates with the selected factors in a selected hospital of Delhi with a view to develop guidelines for the health professionals." Indian journal of palliative care 15.2 (2009): 111.
44. Sörensson, M., et al. "Self‐reported sexual dysfunction in patients with rectal cancer." Colorectal Disease 22.5 (2020): 500-512.
45. Su, Xi, et al. "Effects of Evidence-Based Continuing Care Bundle on Health Outcomes in Rectal Cancer Patients With Temporary Stomas: A Multicenter Randomized Controlled Trial." Cancer nursing 44.3 (2021): 223-234.
46. Sun, Virginia, et al. "Cancer survivors’ challenges with ostomy appliances and self-management: a qualitative analysis." Supportive Care in Cancer 28.4 (2020): 1551-1554.
47. Sun, Virginia, et al. "From diagnosis through survivorship: health-care experiences of colorectal cancer survivors with ostomies." Supportive Care in Cancer 22.6 (2014): 1563-1570.
48. Tejido-Sánchez, A., et al. "Quality of life in patients with ileal conduit cystectomy due to bladder cancer." Actas Urológicas Españolas (English Edition) 38.2 (2014): 90-95.
49. Tong, Guojun, et al. "When do defecation function and quality of life recover for patients with non-ostomy and ostomy surgery of rectal cancer?." BMC surgery 20 (2020): 1-11.
50. van Ginkel, F., et al. "Spinal cord injuries and bowel stomas: timing and satisfaction with stoma formation and alterations in quality of life." Spinal Cord Series and Cases 7.1 (2021): 1-7.
51. Wade, Barbara E. "Colostomy patients: psychological adjustment at 10 weeks and 1 year after surgery in districts which employed stoma‐care nurses and districts which did not." Journal of advanced nursing 15.11 (1990): 1297-1304.
52. Walma, Marieke S., et al. "Predictors of fecal incontinence and related quality of life after a total mesorectal excision with primary anastomosis for patients with rectal cancer." Annals of coloproctology 31.1 (2015): 23.
53. Walming, S., et al. "Quality of life in patients with resectable rectal cancer during the first 24 months following diagnosis." Colorectal Disease 22.12 (2020): 2028-2037.
54. Wang, Meng, Hao Wang, and Wenxian Guan. "Clinical application of artificial anal reconstruction after laparoscopic abdominoperineal resection in low rectal cancer patients." Zhonghua wei chang wai ke za zhi= Chinese journal of gastrointestinal surgery 18.4 (2015): 354-357.
55. Wiltink, Lisette M., et al. "Health-related quality of life 14 years after preoperative short-term radiotherapy and total mesorectal excision for rectal cancer: report of a multicenter randomised trial." European journal of cancer 50.14 (2014): 2390-2398.
56. Zhou, Shu-Ping, et al. "A Prediction Model for Cognitive Impairment Risk in Colorectal Cancer after Chemotherapy Treatment." BioMed Research International 2021 (2021).
57. Harris, Michelle S., Katherine Kelly, and Carol Parise. "Does preoperative ostomy education decrease anxiety in the new ostomy patient? A quantitative comparison cohort study." Journal of Wound Ostomy & Continence Nursing 47.2 (2020): 137-139.
58. Brook, Itzhak, Hans Bogaardt, and Corina van As-Brooks. "Long-term use of heat and moisture exchangers among laryngectomees: medical, social, and psychological patterns." Annals of Otology, Rhinology & Laryngology 122.6 (2013): 358-363.
59. Song, Lili, et al. "Body image in colorectal cancer patients: A longitudinal study." Psycho‐Oncology (2021).
60. Tillin, Therese, et al. "Third‐party prospective evaluation of patient outcomes after dynamic graciloplasty." British journal of surgery 93.11 (2006): 1402-1410.
61. Collaborative, R. E. A. C. C. T. (2022). Post-Operative Functional Outcomes in Early Age Onset Rectal Cancer. *Frontiers in Oncology*, *12*.
62. Gao, J., Wang, H., & Wang, Z. (2022). Study on Ultrasonographic Diagnosis and Postoperative Comprehensive Nursing of Rectal Cancer in Preoperative Staging. *Contrast Media & Molecular Imaging*, *2022*.

**Wrong study type**

1. Akyol, C., et al. "Preconditioning facilitates stoma self care, decreases predisposition to anxiety-depression and improves quality of life in stoma patients: LTP5." Colorectal Disease 14 (2012).
2. Alqahtani, M. A., et al. "CAN HOSPITAL READMISSION FOR DEHYDRATION IN PATIENTS WITH A DIVERTING LOOP ILEOSTOMY BE PREDICTED? A NATIONAL READMISSION DATABASE ANALYSIS." DISEASES OF THE COLON & RECTUM. Vol. 62. No. 6. TWO COMMERCE SQ, 2001 MARKET ST, PHILADELPHIA, PA 19103 USA: LIPPINCOTT WILLIAMS & WILKINS, 2019.
3. Araujo, R. O., et al. "Rectal Cancer after preoperative chemoradiation: analysis of tumor downstaging, sphincter preservation, and quality of life in a randomized study comparing two neoadjuvant regimens." European Journal of Surgical Oncology 45.2 (2019): e45-e46.
4. Armbruster, Shannon Dawn, et al. "Prospective assessment of patient perception and quality of life in gynecologic patients after pelvic exenteration." (2016): 10082-10082.
5. Westney, O., et al. "FACTORS CORRELATING WITH SEXUAL INTEREST AND FUNCTION IN LONG-TERM COLORECTAL CANCER SURVIVORS: 083." The Journal of Sexual Medicine 12 (2015).
6. Bangsgaard, L., et al. "MON-PP120: Quality of Life in the Largest Danish Population of Intestinal Failure Patients Receiving Home Parenteral Nutrition." Clinical Nutrition 34 (2015): S172-S173.
7. Barros, J., et al. "N799 Inflammatory bowel disease affects sexual female desire and sexual female excitement." Journal of Crohn's and Colitis 11.suppl_1 (2017): S491-S491.
8. Bassy, Nadia, and Berit Libutzki. "PCN529 BURDEN OF ILLNESS IN NEW OSTOMATES: AN ANALYSIS BASED ON GERMAN CLAIMS DATA." Value in Health 22 (2019): S540-S541.
9. Behm, K., et al. "A MULTI-INSTITUTIONAL STUDY OF LONG-TERM PATIENT-REPORTED OUTCOMES AFTER PROCTECTOMY VS. PROCTOCOLECTOMY FOR RECTAL CANCER IN PATIENTS WITH LYNCH SYNDROME." DISEASES OF THE COLON & RECTUM. Vol. 59. No. 5. TWO COMMERCE SQ, 2001 MARKET ST, PHILADELPHIA, PA 19103 USA: LIPPINCOTT WILLIAMS & WILKINS, 2016.
10. Benedict, Catherine, et al. "Body Image in Women Following Primary Treatment for Anal and Rectal Cancer: P2–39." Pscyho-oncology 23 (2014): 124-125.
11. Bilali, V., et al. "Psychological attitude to self-perception of stoma patients: P054." Colorectal Disease 16 (2014).
12. Bilgutay, Aylin, Andrew Kirsch, and Michael Garcia-Roig. "ROBOT-ASSISTED LAPAROSCOPIC ILEOVESICOSTOMY: V09-11." The Journal of Urology 199.4 (2018).
13. Brown, C., et al. "P450 Long-term impacts of colectomy surgery among ulcerative colitis patients study (LOCUS): the final analysis." Journal of Crohn's and Colitis 7.Supplement_1 (2013): S190-S190.
14. Bryant, C. C., et al. "Anterior perineal plane for ultra-low anterior resection of the rectum (The APPEAR Technique) clinical, functional and oncological medium term outcomes: P034." Colorectal Disease 14 (2012).
15. Bullen, Tracey, et al. "Body Image Disturbance and the Impact of Surgery Amongst Patients with Colorectal Cancer: A-385." Psycho-oncology 19 (2010).
16. Carter, B. “Juggling symptoms: Dealing with, concealing and underplaying pain by adolescents and young adults with inflammatory bowel disease (IBD).” British Journal of Pain. 14 (1): 5-6 (2020)
17. Chang, E. M., et al. "The Patient’s Perspective on Chemoradiation for Anal Cancer: Evaluation of Expectations and Stigma." International Journal of Radiation Oncology, Biology, Physics 105.1 (2019): E592-E593.
18. Chongpison, Yuda, et al. "Depression and perceived financial burden among long-term rectal cancer survivors." American Psychosocial Oncology Society 11th Annual Conference. 2014.
19. Comelli, Simone, et al. "Intra-arterial infusion of irinotecan loaded microparticles (IAIRIM) for symptomatic and unresectable colorectal cancer (UCRC): Preliminary report." (2013): e20562-e20562.
20. Eardley, A., and SCHOFIELD PF. "Colostomy: the consequences of surgery." (1976).
21. Faury, Stephane, Eric Rullier, and Bruno Quintard. "Body Image, depression and anxiety of rectal cancer patients with temporary stoma: An exploratory longitudinal study." ANNALES MEDICO-PSYCHOLOGIQUES. Vol. 175. No. 10. 21 STREET CAMILLE DESMOULINS, ISSY, 92789 MOULINEAUX CEDEX 9, FRANCE: MASSON EDITEUR, 2017.
22. Faury, S., E. Rullier, and B. Quintard. "What perceptions do men have about temporary stoma after a rectal cancer surgery? An exploratory study." PSYCHO-ONCOLOGIE 11.3 (2017): 158-165.
23. Foster, Claire, et al. "Trajectories of quality of life, health status and personal wellbeing up to two years after curative intent treatment for colorectal cancer: results from the UK ColoREctal Wellbeing (CREW) cohort study." (2016).
24. Foster, Claire, et al. "The impact of primary colorectal cancer treatment on physical symptoms and functioning in the first two years: results from the colorectal well-being (CREW) cohort study." Psycho-Oncology 25.S1 (2016).
25. Foster, C. et al., “Quality of life, health and personal wellbeing up to two years following curative intent colorectal cancer surgery: Results from the UK colorectal wellbeing (CREW) study”. Supportive Care in Cancer 22 (1): S316
26. Geng, Wei, et al. "Continuous nursing reduces postoperative complications and improves quality of life of patients after enterostomies." INTERNATIONAL JOURNAL OF CLINICAL AND EXPERIMENTAL MEDICINE 12.5 (2019): 5895-5901.
27. Green, A., et al. "P458 Sleep problems in inflammatory bowel disease; When bed becomes a battleground." Journal of Crohn's and Colitis 11.suppl_1 (2017): S311-S311.
28. Hagan, Matilda, et al. "Predictors of Re-admissions in Hospitalized Patients With Inflammatory Bowel Disease Treated at a Tertiary Care Hospital: 1748." American Journal of Gastroenterology 109 (2014): S517-S518.
29. Hashash, Jana, et al. "P-028 Predictors of Suicidal Severity Amongst Suicidal IBD Patients." Inflammatory Bowel Diseases 22.suppl_1 (2016): S18-S18.
30. Hashash, Jana G., et al. "Tu1255 Predictors of Depression in Outpatients With IBD." Gastroenterology 148.4 (2015): S-838.
31. HAYRAN KM, Yuce D., et al. "Transcultural Adaptation of EORTC-QLQ-CR29 Scale into Turkish, and Assessment of Validity and Reliability in Turkish Patients with Colorectal Cancer." (2018).
32. He, H., et al. "Effects of a psychosocial intervention programme on improving outcomes of patients with a stoma." European Journal of Cancer 72 (2017): S162.
33. He, H., et al. "P149 Urban life is an independent risk factor for psychological symptoms of the Chinese patients with Crohn's disease." Journal of Crohn's and Colitis 11.suppl_1 (2017): S151-S152.
34. Hedrick, T., et al. "A PILOT STUDY OF PATIENT CENTERED OUTCOME ASSESSMENT FOR PATIENTS UNDERGOING COLORECTAL SURGERY WITHIN AN ENHANCED RECOVERY PROTOCOL." DISEASES OF THE COLON & RECTUM. Vol. 59. No. 5. TWO COMMERCE SQ, 2001 MARKET ST, PHILADELPHIA, PA 19103 USA: LIPPINCOTT WILLIAMS & WILKINS, 2016.
35. Hoogkamer, A., et al. "Prospective study to predict psychological morbidity in young people with inflammatory bowel disease using novel risk assessment tool." JOURNAL OF CROHNS & COLITIS. Vol. 13. GREAT CLARENDON ST, OXFORD OX2 6DP, ENGLAND: OXFORD UNIV PRESS, 2019.
36. Hornbrook, Mark C., et al. "Predictors of SF-6D Scores Among Long-term Colorectal Cancer Survivors." 4th Biennial Cancer Survivorship Research Conference. 2008.
37. Hu, Huan, Jiayu Zheng, and Lei Gao. "The effect of continuing nursing services on colostomy patients." INTERNATIONAL JOURNAL OF CLINICAL AND EXPERIMENTAL MEDICINE 13.8 (2020): 5876-5884.
38. Hubbard, Gill, et al. "HALT (Hernia Active Living Trial): protocol for a feasibility study of a randomised controlled trial of a physical activity intervention to improve quality of life in people with bowel stoma with a bulge/parastomal hernia." Pilot and Feasibility Studies 6.1 (2020): 1-14.
39. Ihn, Myong Hoon, et al. "Cultural adaptation and validation of the Korean version of the EORTC QLQ-CR29 in patients with colorectal cancer." Supportive Care in Cancer 23.12 (2015): 3493-3501.
40. Incrocci, Luca. "Living with Cancer and Sexuality." JOURNAL OF THORACIC ONCOLOGY. Vol. 10. No. 9. 360 PARK AVE SOUTH, NEW YORK, NY 10010-1710 USA: ELSEVIER SCIENCE INC, 2015.
41. Indrebø, Kirsten Lerum, et al. "A new model of patient-reported outcome monitoring with a clinical feedback system in ostomy care: rationale, description and evaluation protocol." Health and quality of life outcomes 18.1 (2020): 1-8.
42. Jayarajah, Umesh, and Dharmabandhu N. Samarasekera. "Psychological wellbeing among patients with intestinal stomas in a tertiary care unit in Sri Lanka." JOURNAL OF GASTROENTEROLOGY AND HEPATOLOGY. Vol. 34. 111 RIVER ST, HOBOKEN 07030-5774, NJ USA: WILEY, 2019.
43. Kan, Olivia, et al. "S0749 Increasing Prevalence and Resource Utilization for Pediatric Inflammatory Bowel Disease Patients With Comorbid Psychiatric Disorders." Official journal of the American College of Gastroenterology| ACG 115 (2020): S378-S379.
44. Kappelman, Michael, et al. "Sexual Interest and Satisfaction in an Internet Cohort of Patients with Inflammatory Bowel Diseases: P‐70." Inflammatory Bowel Diseases 18 (2012): S43-S44.
45. Karimuddin, A., et al. "17 A cross-sectional survey of health and quality of life of patients awaiting colorectal surgery in Canada." Canadian Journal of Surgery 58.4 Suppl 2 (2015).
46. Keller, D. S., et al. "LONG-TERM FUNCTIONAL AND QUALITY OF LIFE OUTCOMES AFTER TATME FOR RECTAL CANCER." DISEASES OF THE COLON & RECTUM. Vol. 61. No. 5. TWO COMMERCE SQ, 2001 MARKET ST, PHILADELPHIA, PA 19103 USA: LIPPINCOTT WILLIAMS & WILKINS, 2018.
47. Keller, M. "Gastrointestinal cancer-Psycho-oncology and quality of life." ZEITSCHRIFT FUR GASTROENTEROLOGIE 38 (2000): 35-40.
48. Khan, M., et al. "THE EFFECTS OF ENDOSCOPIC SEVERITY AND BIOLOGIC TREATMENT ON LEVELS OF DEPRESSION AND DISABILITY AMONGST IBD PATIENTS: A CASE CONTROL STUDY." Gut 62.Suppl 2 (2013): A43-A44.
49. Kittinouvarat, S., M. Sarkarindra, and S. Kittinouvarat. "The study of depressive symptoms in faecal diversion patients." World Council Enterostomal Ther J 22 (2002): 33-37.
50. Kosmach-Park, Beverly. "Outcomes, care needs and adherence in pediatric intestine transplant recipients at 10 or more years post-transplant." PEDIATRIC TRANSPLANTATION. Vol. 23. 111 RIVER ST, HOBOKEN 07030-5774, NJ USA: WILEY, 2019.
51. Kosmach-Park, B., and G. Mazariegos. "Clinical Outcomes, Care Needs and Adherence in Long Term Intestine Transplant Survivors>= 10 yrs." AMERICAN JOURNAL OF TRANSPLANTATION. Vol. 18. 111 RIVER ST, HOBOKEN 07030-5774, NJ USA: WILEY, 2018.
52. Krarup, P., et al. "ANASTOMOTIC LEAK DECREASES QUALITY OF LIFE IN COLON CANCER SURVIVORS: 10 YEARS FOLLOW-UP OF A NATIONWIDE COHORT." DISEASES OF THE COLON & RECTUM. Vol. 61. No. 5. TWO COMMERCE SQ, 2001 MARKET ST, PHILADELPHIA, PA 19103 USA: LIPPINCOTT WILLIAMS & WILKINS, 2018.
53. Lam, Wendy WT, et al. "Supportive Care Needs among Chinese Patients Diagnosed with Colorectal Cancer: A Longitudinal Study." PSYCHO-ONCOLOGY. Vol. 23. 111 RIVER ST, HOBOKEN 07030-5774, NJ USA: WILEY-BLACKWELL, 2014.
54. Latıf, A., F. Shabbır, and Kamran Hameed. "Prevalence of intestinal stoma formation in the management of abdominal surgery in Emergency Department of Allama Iqbal Memorial Teaching Hospital." PJMH S JUN 10 (2016): 498-502.
55. Li, Jing, Xiaoyu Liu, and Jun Chen. "Analyzing Risk Factors for Enterostomy Infection and Neuropsychology of Patients by Computer Information Data Regression under Endoscopic Image Guidance." Neuroscience Letters (2020): 135223.
56. Tau, Loi Tien, and Sally Wai-chi Chan. "Exploring the quality of life and the impact of the disease among patients with colorectal cancer: A systematic review." JBI Evidence Synthesis 9.54 (2011): 2324-2378.
57. Loi, Tien Tau, et al. "Factors predicting health-related quality of life of patients with colorectal câncer one year after surgery." Psycho-oncology. Vol. 23. 111 RIVER ST, HOBOKEN 07030-5774, NJ USA: WILEY-BLACKWELL, 2014.
58. McCawley, A., H. Mannix Jr, and D. D. McCarthy. "The psychological problems of ostomates." Connecticut medicine 39.3 (1975): 151-155.
59. McDermott, E., et al. "P328 Understanding sexual activity and satisfaction in inflammatory bowel disease. Development and validation of a short self-administered assessment tool." Journal of Crohn's and Colitis 7 (2013): S141.
60. McGeer, R., et al. "P372 A pilot study: A psychological support service for patients with inflammatory bowel disease (PSSPIBD)." Journal of Crohn's and Colitis 12.supplement_1 (2018): S292-S293.
61. McGeer, Rona, et al. "OTU-034 IBD psychological support pilot reduces ibd symptoms and improves psychological wellbeing." (2018): A224-A226.
62. Merrick, V., et al. "P125 Rapid increase in pan-treatment refractory Crohn's disease after transition to adult services: a regional cohort study." Journal of Crohn's and Colitis 11.suppl_1 (2017).
63. Moore, Amy K., Karen A. Esquibel, and Wendy Thal. "Ostomy options for clients with ileostomies." Gastroenterology Nursing 31.6 (2008): 418-420.
64. Moussas, G. I., et al. "Psychological and psychiatric problems in cancer patients: relationship to the localization of the disease." Psychiatrike= Psychiatriki 23.1 (2012): 46-60.
65. Mrak, K., and J. Tschmelitsch. "303. Quality of Life After Surgery for Low Rectal Cancer-Pouch Anastomosis Vs Stoma." European Journal of Surgical Oncology 38.9 (2012): 828.
66. Nelson, Ashley, et al. "Experiences Among Survivors of Pelvic Exenteration for Gynecologic Cancer: 25-5." Pscyho-oncology 25 (2016): 70-71.
67. Otsuji-Miwa, Naoko. "QUALITY OF LIFE: PATIENTS WITH SPINAL CORD INJURY WHO ELECT TO UNDERGO COLOSTOMY FOR BOWEL MANAGEMENT." JOURNAL OF WOUND OSTOMY AND CONTINENCE NURSING. Vol. 47. TWO COMMERCE SQ, 2001 MARKET ST, PHILADELPHIA, PA 19103 USA: LIPPINCOTT WILLIAMS & WILKINS, 2020.
68. Perera, Nishan, et al. "Quality of life and anal sphincter manometry following sphincter repair." JOURNAL OF GASTROENTEROLOGY AND HEPATOLOGY. Vol. 34. 111 RIVER ST, HOBOKEN 07030-5774, NJ USA: WILEY, 2019.
69. Qaderi, S., et al. "Follow-up practice and health care consumption during surveillance of colorectal cancer survivors." Diseases of the Colon & Rectum 63.6 (2020): E242-E242.
70. Rethmeier, L. O., E. B. Boisen, and C. Cabral. "Burden of Illness in Ostomates: A German-Based Claims Database Analysis." Value in Health 21 (2018): S83.
71. Rizzola, N. "Psychological attitudes and defensive adjustments in ostomy patients." Minerva psichiatrica 23.1 (1982): 21-25.
72. Rouholiman, Dara, et al. "Improving health-related quality of life of patients with an ostomy using a novel digital wearable device: Protocol for a Pilot Study." JMIR research protocols 7.3 (2018): e82.
73. Rud, C. L., et al. "Quality of life and wellbeing in patients with an ileostomy: A cross sectional study." Clinical Nutrition ESPEN 40 (2020): 514.
74. Pramod, D., P. Vaishali, and A. Nirmalkumar. "ASSESSMENT OF ECONOMICS/QUALITY OF LIFE IN GASTROINTESTINAL-CANCER PATIENTS IN RESOURCE-POOR-SETTINGS: INITIATIVES BY COMMUNITY-CANCER-NGO IN INDIA." ANNALS OF ONCOLOGY. Vol. 20. GREAT CLARENDON ST, OXFORD OX2 6DP, ENGLAND: OXFORD UNIV PRESS, 2009.
75. Schneider, C., H. Grosch, and G. Moeslein. "The continent colostomy: P098." Colorectal Disease 11 (2009).
76. Stokes, Tony, and Sarah Wright. "Body image disturbance and the impact on adjustment pre-surgery amongst colorectal cancer and bowel disease patients." (2009): 117.
77. Solano-Murillo, P., et al. "Health-related quality of life (HRQOL) in colorectal cancer patients who finish their treatment in a tertiary hospital." Value in Health 16.3 (2013): A195.
78. Soliman, Pamela T., et al. "Longitudinal quality of life (QOL) and sexual functioning in women undergoing pelvic exenteration for gynecologic malignancies." (2013): 5608-5608.
79. Tuschhoff, T. "Evaluation of a questionnaire measuring psycho-social burdens of patients with colostomy." ZEITSCHRIFT FUR GASTROENTEROLOGIE 38 (2000): 66-70.
80. Vachon, Ashley, et al. "P-019 YI Risk Factors for Suicidality Amongst Depressed IBD Patients." Inflammatory Bowel Diseases 22.suppl_1 (2016): S15-S15.
81. van Wulfften Palthe, Olivier D., et al. "Analysis of the Sacral Tumor Survey and Recommendation for Future Development." The Spine Journal 16.10 (2016): S320-S321.
82. Wheelwright, Sally, et al. "Representativeness of participants continuing in a five year longitudinal cohort study of colorectal cancer patients receiving curative intent treatment." PSYCHO-ONCOLOGY. Vol. 27. 111 RIVER ST, HOBOKEN 07030-5774, NJ USA: WILEY, 2018.
83. Wheelwright, Sally, et al. "The impact of a stoma on symptoms and functioning following curative surgery for colorectal cancer: Findings from the ColoREctal Wellbeing (CREW) cohort study." PSYCHO-ONCOLOGY. Vol. 28. 111 RIVER ST, HOBOKEN 07030-5774, NJ USA: WILEY, 2019.
84. White, D. et al. “Patient reported outcomes after laparoscopic surgery for rectal cancer”. Surgical Endoscopy 33 (2): S666 (2019)
85. Winter, Jane, et al. "The First Two Years After Colorectal Cancer Treatment: UK Colorectal Wellbeing (CREW) Cohort Study." CANCER NURSING. Vol. 39. TWO COMMERCE SQ, 2001 MARKET ST, PHILADELPHIA, PA 19103 USA: LIPPINCOTT WILLIAMS & WILKINS, 2016
86. Wojewoda, B., et al. "The quality of life patients' with intestinal stoma." ONKOLOGIA POLSKA 9.4 (2006): 184.
87. Xu, Wei, et al. "Clinical efficacy of comprehensive continuing care for early post-discharge patients with colostomy caused by rectal cancer." INTERNATIONAL JOURNAL OF CLINICAL AND EXPERIMENTAL MEDICINE 13.12 (2020): 9963-9969.
88. Yasan, Azia. "Quality of life, depression and anxiety among patients who have undergone permanent or temporary ostomy." (2008).
89. Aldoori, J., Baumber, C., Flannery, A., Holding Brown, F., Jenner, L., & Mockford, K. A. (2021). Incidence of anxiety and depression in long stay colorectal inpatients: A prospective study. *Colorectal Disease*, 61-62.
90. Bianchi, R, Von Känel, R, Roth, R, Schreiner, P, Rossel, JB, Barry, MP, … Biedermann, L 2022, ‘P414 Stoma in patiens with IBD: friend or foe? -\tEffect of stoma on course of disease, psychological well-being and working capacity’, *Journal of Crohn’s and Colitis*, vol. 16, no. Supplement_1, pp. i404–i406.
91. Blackwell, J., Saxena, S., Jayasooriya, N., Petersen, I., Hotopf, M., Creese, H., ... & POP-IBD study group. (2022). Stoma formation in Crohn’s Disease and the likelihood of antidepressant use: a population-based cohort study. *Clinical Gastroenterology and Hepatology*, *20*(4), e703-e710. [already included in study; appeared multiple times across two searches as article was in press]
92. Blackwell, J., Saxena, S., Jayasooriya, N., Alexakis, C., Hotopf, M., & Pollok, R. (2021). P86 Permanent stoma formation in Crohn’s disease is associated with increased rates of antidepressant use. *Gut*, *70*(Suppl 1), A85-A85.
93. Cooney, R., Barrett, K., & Russell, R. K. (2023). P822 The negative impact of mental health comorbidity in children and young adults with inflammatory bowel disease: a UK population-based cohort study. *Journal of Crohn's and Colitis*, *17*(Supplement_1), i956-i957.
94. Fernández Candela, A., Sánchez-Guillén, L., García Catalá, L., Curtis Martínez, C., Bosch Ramírez, M., Lario Pérez, S., ... & Arroyo Sebastián, A. (2021). IMPACT OF LAPAROSCOPIC COLORECTAL SURGERY ON BODY IMAGE. *British Journal of Surgery*, *108*(Supplement_3), znab160-023.
95. Fernández Candela, A., Sánchez-Guillén, L., García Catalá, L., Curtis Martínez, C., Bosch Ramírez, M., Lario Pérez, S., ... & Arroyo Sebastián, A. (2021). IMPACT OF LAPAROSCOPIC COLORECTAL SURGERY ON BODY IMAGE. *Surgical Endoscopy*, 36 (2): S491-S492
96. Ferreira, A., Sardinha, M., Baleiras, M., Neves, M., Matos, A., Malheiro, M., ... & Martins, A. (2021). P-1 Health-related quality of life of rectal cancer survivors treated with curative intent. *Annals of Oncology*, *32*, S97.
97. Ji, U., & Kim, H. (2022, May). RISK OF DEPRESSIVE DISORDER OF RECTAL CANCER PATIENTS WITH STOMA: NATIONWIDE POPULATION-BASED COHORT STUDY. In *DISEASES OF THE COLON & RECTUM* (Vol. 65, No. 5, pp. 243-244). TWO COMMERCE SQ, 2001 MARKET ST, PHILADELPHIA, PA 19103 USA: LIPPINCOTT WILLIAMS & WILKINS.
98. Karamanliev, Dimitrov, 2022. Quality of life after transanal total mesorectal excision in rectal cancer patients -A single-center initial experience. *Colorectal Disease*, 24: 145-146
99. Ketterer, S. N., Leach, M. J., & Fraser, C. (2021). Factors associated with quality of life among people living with a stoma in nonmetropolitan areas. *Nursing Research*, *70*(4), 281. [[already included in study; appeared multiple times across two searches as article was in press]
100. Khosla, D., Rana, S., Kapoor, R., Periasamy, K., Madan, R., Kumar, N., & Gupta, R. (2021). P-243 A prospective study to assess the impact of colostomy on psychosocial aspects of quality of life in colorectal cancer patients. *Annals of Oncology*, *32*, S181-S182.
101. Melliti, R., Ammar, N., Souilem, A., Sabrine, N., Bouzaabia, N., Chabchoub, I., ... & Ahmed, S. B. (2021). P-270 Impact of intestinal stoma on quality of life in Tunisian patients with colorectal cancer. *Annals of Oncology*, *32*, S189-S190.
102. Moulton et al, 2023. Using antidepressants for refractory symptoms of Inflammatory Bowel Disease: A prospective service evaluation, *Journal of Crohn’s and Colitis*, 17: i538-i539
103. Patel, A., Joshi, H., Wagh, A., & Bhatt, C. (2022, January). To study the prevalence of depression and anxiety in patients of Inflammatory Bowel Disease (IBD) and the efficacy of psychopharmacotherapy in these patients: A pilot study. In *JOURNAL OF CROHNS & COLITIS* (Vol. 16, pp. I389-I389). GREAT CLARENDON ST, OXFORD OX2 6DP, ENGLAND: OXFORD UNIV PRESS.
104. Ssewanyana, Y., Ssekitooleko, B., Suuna, B., Bua, E., Wadeya, J., Makumbi, T. K., ... & Omona, K. (2021). Quality of life of adult individuals with intestinal stomas in Uganda: a cross sectional study. *African health sciences*, *21*(1), 427-36. [already included in study; appeared multiple times across two searches as article was in press]
105. Tang, W. S. W., Chiang, L. L. C., Kwang, K. W., & Zhang, M. W. B. (2022). Prevalence of depression and its potential contributing factors in patients with enterostomy: A meta-analytical review. *Frontiers in Psychiatry*, *13*.
106. Van der Storm, 2022. Stoma-APPtimize: improving the self-efficacy and supporting self-confidence of patients having a stoma using an evidence-based, mobile app, *Surgical Endoscopy*, 36 (2): S368-S369

**Data of interest unable to be extracted**

1. Ackerstaff, A. H., et al. "Heat and moisture exchangers as a treatment option in the post‐operative rehabilitation of laryngectomized patients." Clinical Otolaryngology & Allied Sciences 20.6 (1995): 504-509.
2. Brown, Sarah R., et al. "The impact of postoperative complications on long-term quality of life after curative colorectal cancer surgery." Annals of surgery 259.5 (2014): 916-923.
3. Dehvan, Fazel, et al. "Predicting the self-efficacy of patients with stoma based on general health." Koomesh 21.1 (2019): 123-130.
4. Digennaro, Rosa, et al. "Coloanal anastomosis or abdominoperineal resection for very low rectal cancer: what will benefit, the surgeon's pride or the patient's quality of life?." International journal of colorectal disease 28.7 (2013): 949-957.
5. Foster, Claire, et al. "Pre-surgery depression and confidence to manage problems predict recovery trajectories of health and wellbeing in the first two years following colorectal cancer: results from the CREW cohort study." PLoS One 11.5 (2016): e0155434.
6. Frankland, Jane, et al. "Prevalence and predictors of poor sexual well-being over 5 years following treatment for colorectal cancer: results from the ColoREctal Wellbeing (CREW) prospective longitudinal study." BMJ open 10.11 (2020): e038953.
7. Foster, Claire, et al. "Trajectories of quality of life, health and personal wellbeing up to two years following curative intent treatment for colorectal cancer: results from the UK Colorectal Wellbeing (CREW) cohort study." European Journal of Cancer Care 24 (2015).
8. Huang, Weidong, et al. "Assessing health-related quality of life of patients with colorectal cancer using EQ-5D-5L: a cross-sectional study in Heilongjiang of China." BMJ open 8.12 (2018): e022711.
9. Gapinska, Bozena, et al. "Self-efficacy and anxiety and depression in patients with colostomy." Wspolczesna Onkologia 12.2 (2008): 84.
10. Iqbal, Atif, et al. "Readmission after elective ileostomy in colorectal surgery is predictable." JSLS: Journal of the Society of Laparoendoscopic Surgeons 22.3 (2018).
11. Jiao H, Wang H, Wang L, Zou L. Original article humanistic care improves negative emotions and quality of life in colon cancer patients undergoing colostomies. International journal of clinical and experimental medicine 2020; 13(8): 6107‐6114.
12. Keyes, Kathryn, et al. "Age differences in coping, behavioral dysfunction and depression following colostomy surgery." The Gerontologist 27.2 (1987): 182-184.
13. Klopp, A. (1990). Body image and self-concept among individuals with stomas. Journal of Enterostomal Therapy, 17 (3), 98-105.
14. Knowles, Simon R., et al. "Exploration of health status, illness perceptions, coping strategies, psychological morbidity, and quality of life in individuals with fecal ostomies." Journal of Wound, Ostomy and Continence Nursing 44.1 (2017): 69-73.
15. Küchenhoff, J., et al. "Coping with a Stoma–A Comparative Study of Patients with Rectal Carcinoma or Inflammatory Bowel Diseases." Psychotherapy and psychosomatics (1981): 98-104.
16. Lee, Michelle WK, et al. "Quality of life, anxiety and depression levels of Chinese stoma patients in Hong Kong." World Council of Enterostomal Therapists Journal 36.1 (2016): 28.
17. Liu, Y. et al. “The influence of peer support education on the effect of postoperative continued care for patients with rectal cancer stoma.” World Chinese Journal of Digestion 23.20 (2015): 3321-3325
18. Mitchell, Kimberly A., et al. "Demographic, clinical, and quality of life variables related to embarrassment in veterans living with an intestinal stoma." Journal of Wound Ostomy & Continence Nursing 34.5 (2007): 524-532.
19. Ross, Lone, et al. "Quality of life of Danish colorectal cancer patients with and without a stoma." Supportive Care in Cancer 15.5 (2007): 505-513.
20. Yanzi and Ying. “The effect of acupoint massage on negative emotions and quality of life in patients with rectal cancer and colostomy.” Integrated Traditional Chinese and Western Medicine Nursing 4.1 (2018): 67.
21. Ye, Xinmei, et al. "Application value of nursing intervention combined with early nutritional support in preventive stoma reversion of low rectal cancer." Oncology letters 17.4 (2019): 3777-3782.
22. Al-Aamri, H., Al-Huseini, S., Chan, M. F., Al Saadi, A., Al-Sibani, N., Al-Dughaishi, Z., & Al-Alawi, M. (2022). Clinical predictors of depression among patients with inflammatory bowel diseases: a cross-sectional analytical study from Oman. *Oman Medical Journal*, *37*(2), e352.
23. Antoniadis, D., Giakoustidis, A., Paramythiotis, D., Michalopoulos, A., Mandanas, Z. N., & Papadopoulos, V. N. (2022). Mental health well-being and functional adjustment in colorectal cancer patients: a prospective cohort study. *Acta Chirurgica Belgica*, 1-8.
24. Bianchi, R., Mamadou-Pathé, B., von Känel, R., Roth, R., Schreiner, P., Rossel, J. B., ... & or the Swiss IBD cohort study. (2022). Effect of closed and permanent stoma on disease course, psychological well-being and working capacity in Swiss IBD cohort study patients. *Plos one*, *17*(9), e0274665.
25. Fuschi, A., Al Salhi, Y., Sequi, M. B., Velotti, G., Martoccia, A., Suraci, P. P., ... & Pastore, A. L. (2021). Evaluation of functional outcomes and quality of life in elderly patients (> 75 yo) undergoing minimally invasive radical cystectomy with single stoma ureterocutaneostomy vs. Bricker intracorporeal ileal conduit urinary diversion. *Journal of Clinical Medicine*, *11*(1), 136.
26. Hu, Z., Zhang, H., Wang, J., Xiong, H., Liu, Y., Zhu, Y., ... & Tang, Q. (2022). Nomogram to predict the risk of postoperative anxiety and depression in colorectal cancer patients. *International Journal of General Medicine*, 4881-4895.
27. Li, L., Liu, L., Kang, H., & Zhang, L. (2021). The influence of predictive nursing on the emotions and self-management abilities of post-colostomy rectal cancer patients. *American Journal of Translational Research*, *13*(6), 6543.
28. Liu, Y., & Ni, L. (2021). Construction and Application of Remote Continuing Care Model for Colorectal Cancer Patients in the Internet Era. *Indian Journal of Pharmaceutical Sciences*, 177-181.
29. Mohamed, N. E., Shah, Q. N., Kata, H. E., Sfakianos, J., & Given, B. (2021, February). Dealing with the unthinkable: bladder and colorectal cancer patients’ and informal caregivers’ unmet needs and challenges in life after ostomies. In *Seminars in oncology nursing* (Vol. 37, No. 1, p. 151111). WB Saunders.
30. Orive, M., Anton-Ladislao, A., Lázaro, S., Gonzalez, N., Bare, M., Fernandez de Larrea, N., ... & REDISSEC-CARESS/CCR group. (2022). Anxiety, depression, health-related quality of life, and mortality among colorectal patients: 5-year follow-up. *Supportive Care in Cancer*, *30*(10), 7943-7954.
31. Oyama, H., Moroi, R., Tarasawa, K., Shimoyama, Y., Naito, T., Sakuma, A., ... & Masamune, A. (2022). Depression is associated with increased disease activity in patients with ulcerative colitis: A propensity score‐matched analysis using a nationwide database in Japan. *JGH Open*.
32. Pan, P., Chen, L., Zhang, D., Rao, S., Tao, Y., & Fan, L. (2022). Continuing Care Bundle in Elderly Patients with Rectal Cancer after Radical Resection with Permanent Stoma. *Evidence-Based Complementary and Alternative Medicine*, *2022*.
33. Qaderi, S. M., Ezendam, N. P., Verhoeven, R. H., Custers, J. A., de Wilt, J. H., & Mols, F. (2021). Follow‐up practice and healthcare utilisation of colorectal cancer survivors. *European Journal of Cancer Care*, *30*(5), e13472.
34. Révész, D., van Kuijk, S. M., Mols, F., van Duijnhoven, F. J., Winkels, R. M., Kant, I., ... & Bours, M. J. (2022). External validation and updating of prediction models for estimating the 1-year risk of low health-related quality of life in colorectal cancer survivors. *Journal of Clinical Epidemiology*, *152*, 127-139.
35. Song, L., Pang, Y., Zhang, J., & Tang, L. (2021). Body image in colorectal cancer patients: A longitudinal study. *Psycho‐Oncology*, *30*(8), 1339-1346.
36. Yu, S., & Tang, Y. (2021). Effects of comprehensive care on psychological emotions, postoperative rehabilitation and complications of colorectal cancer patients after colostomy. *American journal of translational research*, *13*(6), 6889.

**Full-text unavailable**

1. Canetrari, R, Ricci Bitti, P. E.. “Psychologic reactions to total colectomy” Medicina Psicosomatica 2 (3): 187-195 (1975)
2. Fryc-Martyńska, J. "Psychological sequelae of abdominal stoma and the value of informing the patients and their families." Polski tygodnik lekarski (Warsaw, Poland: 1960) 44.20 (1989): 469-471.
3. Glinska, J. “Intestinal stoma and emotional state. Methodology of nurses' work and emotional support of patients” Proktologia 8 (2): 129-136 (2007)
4. Granados-García, J., et al. "Retrospective study of 50 ileostomies at the Salvador Zubirán National Institute of Nutrition." Revista de investigacion clinica; organo del Hospital de Enfermedades de la Nutricion 48.2 (1996): 111-115.
5. Hadjittofi, C., et al. “Late ileostomy closure does not affect long-term bowel function after rectal cancer surgery”. Colorectal Disease 20: 6 (2018)
6. Huiying, Q. “Investigation and analysis of the psychological status among patients with permanent colostomies”. 38 (4): S68 (2015)
7. Kadam, P. et al. “Service innovation audit on psychological support of stoma patients: Phase one.” Colorectal Disease 21: 51 (2019)
8. Kang, L. et al. “Survey of stigma of rectal cancer patients with permanent colostomy and its influencing factors.” Chinese Nursing Research 31 (28): 3527-3530 (2017)
9. Lam, C. et al. “Quality of life and burden of care in colorectal cancer: A study on patients and caregivers.” Cancer Nursing 38 (4): S23-S24 (2015)
10. Ma, J. et al. “Influence of anastomosis level on defection and life quality of patients underwent sphincter preservation for rectal cancer” World Chinese Journal of Digestology 17 (2): 221-224 (2009)
11. Oliveira, A. et al. “Feelings about the stoma and its implications for the quality of life Ostomates” European Journal of Epidemiology 27 (1): S178 (2012)
12. Rajkumar, K. et al. “Psychosocial issues after pelvic exenteration” Indian Journal of Surgical Oncology 4 (2): 212 (2013)
13. Wronska, L., Wiraszka, G. “Quality of life of patients with colorectal cancer and with stoma” Psychooncology / Psychoonkologia 7 (2): 37-42 (2003)
14. Al-Amri et al, 2022. Prevalence of Depression among Patients with Infammatory Bowel Diseases Admitted to SQUH, *Oman Medical Journal*, 37: 1

**APPENDIX 3 = SUPPLEMENTARY FIGURES**

**Supplementary Figure 1. Funnel plot summarising publication bias for studies with data regarding effect of age on depressive symptoms after stoma surgery**

**Supplementary Figure 2. Funnel plot summarising publication bias for studies with data regarding mean age and standard deviations in studies with data regarding effect of age on depressive symptoms after stoma surgery**

**Supplementary Figure 3. Funnel plot summarising publication bias for studies with data regarding effect of sex on depressive symptoms after stoma surgery**

**Supplementary Figure 4. Forest plot summarising prevalence of male sex in studies with data regarding effect of sex on depressive symptoms after stoma surgery**

**Supplementary Figure 5. Funnel plot summarising publication bias for studies with data regarding prevalence of male sex and data regarding effect of sex on depressive symptoms after stoma surgery**

**Supplementary Figure 6. Forest plot showing prevalence of depressive symptoms after stoma surgery in studies reporting by region**

**Supplementary Figure 7. Funnel plot summarising publication bias for studies with data regarding effect of region on depressive symptoms after stoma surgery**

**Supplementary Figure 8. Forest plot summarising the odds of experiencing depressive symptoms before versus after stoma surgery**

**Supplementary Figure 9. Funnel plot summarising publication bias in studies reporting odds of experiencing depressive symptoms before versus after stoma surgery**

**Supplementary Figure 10. Forest plot summarising the odds of experiencing depressive symptoms after surgery in stoma versus non-stoma populations**

**Supplementary Figure 11. Funnel plot summarising publication bias in studies reporting data regarding the odds of experiencing depressive symptoms after surgery in stoma versus non-stoma populations**

**Supplementary Figure 12. Forest plot summarising mean differences in experiencing depressive symptoms after surgery in stoma versus non-stoma populations**

**Supplementary Figure 13. Funnel plot summarising publication bias in studies reporting data regarding mean differences in experiencing depressive symptoms after surgery in stoma versus non-stoma populations**

**Supplementary Figure 14. Forest plot summarising the mean differences in experiencing depressive symptoms after surgery in colostomy versus ileostomy patients**

**Supplementary Figure 15. Funnel plot summarising publication bias in studies reporting data regarding the mean differences in experiencing depressive symptoms after surgery in colostomy versus ileostomy patients**

| First author | Year | REPORTING Average Reviewer A+B (/11) | EXTERNAL VALIDITY Average Reviewer A+B (/3) | BIAS Average Reviewer A+B (/7) | CONFOUNDING Average Reviewer A+B (/6) | POWER Average Reviewer A+B (/5) | TOTAL Average Reviewer A+B (/32) |
| --- | --- | --- | --- | --- | --- | --- | --- |
| Abdalla | 2016 | 9.5 | 0.5 | 4.5 | 2.5 | 5 | 19.5 |
| Ananthakrishnan | 2013 | 8 | 3 | 4 | 2.5 | 5 | 20 |
| Anaraki | 2012 | 9.5 | 1.5 | 3 | 2.5 | 5 | 19 |
| Anaraki | 2012 | 9.5 | 1.5 | 4 | 3 | 5 | 20.5 |
| Armbruster | 2018 | 10 | 0.5 | 4 | 2.5 | 5 | 19.5 |
| Bahayi | 2018 | 8.5 | 1 | 3.5 | 2 | 5 | 17.5 |
| Barisic | 2011 | 7.5 | 0.5 | 4.5 | 1.5 | 2 | 15 |
| Bau | 2001 | 7 | 0 | 4.5 | 0.5 | 5 | 14.5 |
| Blackwell | 2020 | 8 | 3 | 5 | 2.5 | 5 | 21 |
| Bossema | 2011 | 9 | 0.5 | 5 | 2.5 | 5 | 19.5 |
| Bullen | 2012 | 10 | 0.5 | 5 | 3 | 5 | 21 |
| Chen | 2013 | 7.5 | 1 | 4.5 | 2.5 | 5 | 18 |
| Coggrave | 2012 | 8.5 | 1 | 4 | 2.5 | 5 | 18.5 |
| Colquhoun | 2006 | 8 | 0 | 4 | 1.5 | 5 | 16 |
| Cotrim | 2008 | 9.5 | 1 | 5 | 2.5 | 5 | 20.5 |
| Davidson | 2016 | 6.5 | 0 | 4 | 2.5 | 2 | 14 |
| Davis | 2020 | 9.5 | 1.5 | 5 | 3 | 5 | 21.5 |
| Geng | 2017 | 10 | 1.5 | 5 | 3.5 | 5 | 22.5 |
| González | 2016 | 7.5 | 2 | 4 | 2.5 | 5 | 18.5 |
| Grant | 2011 | 5.5 | 0 | 4 | 2.5 | 1 | 12.5 |
| Holzer | 2005 | 9 | 0.5 | 4 | 2.5 | 5 | 18.5 |
| Hong | 2014 | 10.5 | 2 | 4 | 3 | 5 | 22 |
| Hornbrook | 2011 | 8.5 | 1 | 4.5 | 3.5 | 5 | 20 |
| Iqbal | 2018 | 8 | 2 | 3.5 | 2.5 | 2 | 17 |
| Jayarajah | 2017 | 9 | 1 | 4.5 | 3 | 5 | 20 |
| Jayarajah | 2017 | 8.5 | 1 | 4 | 3.5 | 5 | 19.5 |
| Jin | 2019 | 7.5 | 0.5 | 4 | 2.5 | 5 | 17 |
| Karakayali | 2016 | 9 | 0.5 | 4 | 2.5 | 5 | 18.5 |
| Keltikangas-Järvinen | 1983 | 5.5 | 0 | 4 | 0.5 | 5 | 12.5 |
| Keltikangas-Järvinen | 1984 | 7.5 | 1.5 | 4 | 2 | 5 | 17.5 |
| Ketterer | 2021 | 9 | 1 | 4.5 | 3.5 | 5 | 20.5 |
| Knowles | 2013 | 7.5 | 0 | 4.5 | 2 | 5 | 16.5 |
| Knowles | 2013 | 7.5 | 1.5 | 4 | 2.5 | 5 | 18 |
| Knowles | 2014 | 7 | 0 | 4.5 | 2.5 | 5 | 16.5 |
| Krouse | 2009 | 9 | 0.5 | 5 | 2.5 | 5 | 19.5 |
| Krouse | 2016 | 9.5 | 0.5 | 4.5 | 2 | 5 | 19 |
| Lamb | 2019 | 6.5 | 0 | 3.5 | 1.5 | 5 | 14 |
| Liu | 2015 | 8 | 1.5 | 5 | 3.5 | 5 | 20.5 |
| Lowe | 2019 | 8.5 | 1 | 4 | 2.5 | 5 | 18.5 |
| MacDonald | 1985 | 8 | 1 | 4 | 2.5 | 5 | 18 |
| Mohamed | 2021 | 8 | 0 | 3.5 | 2.5 | 5 | 16.5 |
| Mols | 2014 | 8.5 | 1 | 5 | 3 | 5 | 20 |
| Norton | 2005 | 9 | 0 | 4.5 | 0.5 | 5 | 16.5 |
| Park | 2018 | 10 | 0 | 3.5 | 3 | 5 | 19 |
| Portier | 2005 | 7.5 | 0 | 5 | 2 | 5 | 17 |
| Powell-Chandler | 2020 | 9.5 | 1 | 4.5 | 3 | 5 | 20.5 |
| Rafiei | 2017 | 8 | 1 | 4 | 3 | 5 | 18.5 |
| Rafiei | 2019 | 7.5 | 1 | 4 | 2.5 | 5 | 17.5 |
| Ramer | 1992 | 7.5 | 0 | 5 | 1 | 2 | 14.5 |
| Reese | 2014 | 9.5 | 0.5 | 4 | 3.5 | 5 | 20 |
| Repić | 2018 | 9 | 1 | 4 | 3 | 5 | 19.5 |
| Richbourg | 2007 | 8.5 | 0.5 | 4 | 2.5 | 3 | 17 |
| Sceats | 2020 | 9 | 3 | 4 | 3 | 5 | 21.5 |
| Sharpe | 2011 | 9 | 1.5 | 4.5 | 3 | 5 | 20.5 |
| Song | 2020 | 9.5 | 0.5 | 4.5 | 3 | 5 | 20 |
| Ssewanyana | 2021 | 9 | 1.5 | 4.5 | 3 | 5 | 20.5 |
| Thomas | 1987 | 5.5 | 2 | 5 | 2 | 5 | 17 |
| Wang | 2018 | 9.5 | 1.5 | 5 | 2.5 | 5 | 21 |
| Williams | 1983 | 7.5 | 1 | 4 | 1.5 | 5 | 16.5 |
| Wirsching | 1975 | 4.5 | 0 | 3.5 | 0.5 | 5 | 11 |
| Leminski | 2021 | 10.5 | 2 | 4 | 2.5 | 5 | 24 |
| Rud | 2022 | 8 | 2 | 4.5 | 3 | 5 | 22.5 |
| Shrestha | 2022 | 9.5 | 2 | 5 | 4 | 5 | 25.5 |
| Sivero | 2022 | 8 | 2 | 4 | 3.5 | 5 | 22.5 |
| Zewude | 2020 | 10.5 | 2 | 4 | 4 | 5 | 25.5 |

**Supplementary Table 1. Risk of bias assessment using the Downs and Black Checklist for 65 non-randomised studies of interventions included in the present systematic review**

| **Article** | **Reviewer A** | **Reviewer B** |
| --- | --- | --- |
| Chaudhri et al (2005) | Low | Some concerns |
| Koc et al (2022) | Low | Some concerns |
| Lim et al (2019) | Low | Some concerns |

**Supplementary Table 2. Overall risk of bias assessment using the Cochrane RoB 2.0 checklist for three randomised controlled trials included in the present systematic review**
